# Novel Natural Candidates for Replacing Synthetic Additives in Nutraceutical and Pharmaceutical Areas: Two *Senna* Species (*S. alata* (L.) Roxb. and *S. occidentalis* (L.) Link)

**DOI:** 10.1002/fsn3.4705

**Published:** 2024-12-22

**Authors:** Sakina Yagi, Mehmet Veysi Cetiz, Gokhan Zengin, Kassim Bakar, Azali Ahamada Himidi, Andilyat Mohamed, Marijana Skorić, Jasmina Glamočlija, Uroš Gašić

**Affiliations:** ^1^ Department of Botany, Faculty of Science University of Khartoum Khartoum Sudan; ^2^ Department of Medical Biochemistry, Faculty of Medicine Harran University Sanliurfa Turkey; ^3^ Department of Biology, Science Faculty Selcuk University Konya Turkey; ^4^ Laboratoire Aliments, Réactivité et Synthèse Des Substances Naturelles, Faculté Des Sciences et Techniques Université Des Comores Moroni Comoros; ^5^ Herbier National Des Comores, Faculté Des Sciences et Techniques Université Des Comores Moroni Comoros; ^6^ Department of Plant Physiology, Institute for Biological Research “Siniša Stanković” – National Institute of Republic of Serbia University of Belgrade Belgrade Serbia

**Keywords:** anthraquinones, bacterial enzymes, molecular docking, radical scavenging, *Senna* species

## Abstract

*Senna alata* (L.) Roxb. and *Senna occidentalis* (L.) Link (family Fabaceae) are commonly used in different systems of traditional medicine to treat ailments. The present study was designed to determine the phytoconstituents, antioxidant, enzyme inhibition, and antimicrobial activities of the methanolic extract from the leaves of these two *Senna* species. A total of 75 phenolic compounds belonging to dihydroxybenzoic acids, dihydroxycinnamic acids, flavonoid C‐glycosides, flavonoid O‐glycosides, flavonoid aglycones, anthraquinone glycosides, and anthraquinone aglycones were identified. Flavonoid C‐glycosides were only found in *S. occidentalis* while sennosides A, B, and C were only detected in *S. alata* . In line with its higher total phenolic and flavonoids contents, *S. alata* exerted significantly (*p* < 0.05) higher antiradical (2,2‐diphenyl‐1‐picrylhydrazy (DPPH) = 58.36 mg trolox equivalent (TE)/g; 2,2′‐azino‐bis(3‐ethylbenzothiazoline‐6‐sulfonic acid (ABTS) = 118.86 mg TE/g), ions reducing (cupric reducing antioxidant capacity (CUPRAC) = 93.85 mg TE/g; ferric reducing antioxidant power (FRAP) = 50.42 mg TE/g), and total antioxidant (1.39 mmol TE/g) activities than *S. occidentalis* . *S. alata* revealed significantly (*p* < 0.05) higher inhibitory effect against butyrylcholinesterase (1.67 mg galantamine equivalent (GALAE)/g), tyrosinase (45.07 mg KAE/g) 45.07 mg kojic acid equivalent (KAE)/g), α‐glucosidase (0.73 mmol acarbose equivalent (ACAE)/g), and α‐amylase (2.95 mmol ACAE/g) enzymes. Both species showed high antibacterial and antifungal activities with remarkable antifungal activity exerted by *S. alata* against *Trichoderma viride* (minimum inhibition concentration (MIC) 1 mg/mL), similar to that of Ketoconazole. The study utilized molecular docking, molecular mechanics Poisson–Boltzmann surface area (MM/PBSA) free energy calculations, and molecular dynamics simulations to evaluate the binding interactions between anthraquinone glycosides and various bacterial enzymes, including targets from *Escherichia coli* and *Staphylococcus aureus* . The findings suggest that compounds like sennoside A, sennoside B, and chrysophanol exhibit strong binding affinities, stable interactions, and potential as antimicrobial inhibitors, especially against vital bacterial proteins such as MurE and 30S ribosome S3. In conclusion, our findings underscore the biopharmaceutical potential of these two *Senna* species, suggesting their significance as sources of bioactive agents for health‐related applications.

## Graphical abstract

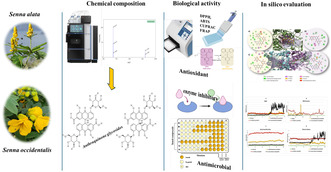

## 1 Introduction

There is continuous interest in exploring plant‐based alternative solutions for humans' welfare. Indeed, medicinal plants have served as the leading source of curative therapies and novel bioactive compounds that are crucial to drug discovery (Abdallah et al. 2023; Ak et al. 2021; Uysal et al. 2018; Yerlikaya et al. 2017). The genus *Senna* (Family Fabaceae) comprises about 250–300 species distributed worldwide. They are annual or biannual herbs, shrubs, or trees found grown in African, Asia, Australia, Europe, and America (Oladeji, Adelowo, and Oluyori 2021). Some *Senna* species are used in traditional medicine since ancient times to treat various diseases. The most popular species is *Senna alexandrina* Mill. (Syn. *Cassia senna* L. and *Cassia acutifolia* Del) which is used to treat constipation, flatulence, diabetes, and skin diseases (Muhakr et al. 2024; Omer et al. 2022). *Senna* species are rich in anthraquinones, alkaloids, phenols, terpenes, and steroids (Alshehri et al. 2022; Oladeji, Adelowo, and Oluyori 2021). Pharmacological studies have shown that they possess laxative (Santos‐Jasso et al. 2017), antimicrobial (Tatsimo et al. 2017), antigonorrhea (Malmir et al. 2015), antiprotozoal (Lim et al. 2018), antinociceptive (Hishe et al. 2018), antioxidant (Afify and Hassan 2016), and anti‐inflammatory (da Silva et al. 2012) activities among others.

Other examples of *Senna* species that are commonly used in different systems of traditional medicine are *S. alata* (commonly known as candle bush or ringworm bush) and *S. occidentalis* (known as coffee senna). Both species are shrubs widely distributed in Asia, Africa, America, and Australia (Oladeji, Adelowo, and Oluyori 2021). *S. alata* is an ornamental and medicinal plant used to cure diverse ailments like constipation, wounds, eczema, diabetes, ringworms, flu, and malaria (Oladeji et al. 2020). *S. occidentalis* are used to treat diseases like diabetes, menstrual problems, filarial disease, liver complaints, anemia, fever, tuberculosis, and fever (Nde et al. 2022). Antraquinones, flavonoids, terpenes, and steroids were identified in the two species (Mahanthesh, Manjappa, Sherikar, et al. 2019; Nde et al. 2022). Pharmacological studies indicated that the two species possess antimicrobial, antioxidant, antidiabetic, dermatophytic, anthelmintic, and antimalarial activities among others (Fatmawati, Purnomo, and Bakar 2020; Nde et al. 2022). In vivo toxicological studies showed that the leaves of the two species did not display any toxic or lethal effect and only pure anthraquinone compounds isolated from *S. alata* revealed subtle hepatorenal toxicity (Panigrahi et al. 2014, 2015; Patel et al. 2014; Yagi, Tigani, and Adam 1998).

In silico methodologies have become an indispensable tool in modern drug discovery and natural product research due to their ability to predict and analyze molecular interactions efficiently (Sliwoski et al. 2014; Trott and Olson 2010). These methodologies, which include molecular docking and molecular dynamics (MD) simulations, permit researchers to screen and evaluate bioactive compounds for their interaction with target proteins, thereby reducing the time and costs associated with experimental procedures (Morris and Lim‐Wilby 2008; Salmaso and Moro 2018). In particular, the study of plant‐derived compounds stands to gain considerably from these approaches, given that these molecules are often structurally complex, diverse, and capable of interacting with multiple biological targets (Cetiz et al. 2024; Cusumano et al. 2024). Computational tools facilitate not only the prediction of binding affinities and interaction mechanisms but also insights into the stability and pharmacokinetic properties of these compounds. This enables the identification of lead molecules for further development (Abraham et al. 2015).

Today, there is a growing interest in *Senna* species due to their wide range of phytoconstituents and pharmacological functions. Comprehensive studies on *S. alata* and *S. occidentalis* were relatively limited and due to their wide utilization in traditional medicine, it is necessary to highlight their phytoconstituents and associated biological activities. Thus, the objective of the present study was to thoroughly investigate the phytochemical profiles, antioxidant, enzyme inhibition, and antimicrobial activities of *S. alata* and *S. occidentalis* leaves. The present study aims to provide a comprehensive chemical and biological characterization of *S. alata* and *S. occidentalis* leaves, with a particular focus on their phenolic content, antioxidant, antimicrobial, and enzyme inhibition activities. Moreover, this study utilizes sophisticated in silico techniques, including molecular docking, MM/PBSA free energy calculations, and MD simulations, to elucidate the interaction mechanisms of key bioactive compounds with biological targets. By integrating experimental and computational methodologies, this research aims to identify potential therapeutic agents, particularly for antibacterial and enzyme inhibition applications.

## 2 Materials and Methods

### 2.1 Plant Materials

*Senna alata* (L.) Roxb. and *S. occidentalis* (L.) Roxb. were collected in Itsinkoudi (Village located in the North‐East of Grande‐Comore) in September 2020 [GPS coordinates: S 11,35109°/E 43,23237°]. The plants were sent to the Comoros national herbarium (University of the Comoros, Moroni, Comoros) for authentication and reference samples were kept in the herbarium (Voucher no: AND‐HKM 440 for *S. alata* and AND‐HKM 442 for *S. occidentalis* ). The leaves were dried at room temperature for 1 week, and then they were ground using a laboratory mill. The powdered plant materials were stored in dark conditions at 4°C.

#### 2.1.1 Plant Extract Preparation

Methanol was used as a solvent in the extraction procedure. Each 10 g sample was macerated with 200 mL of methanol for 24 h at ambient temperature. Methanol was removed via evaporation under low pressure, and the extracts were kept at 4°C until analysis.

#### 2.1.2 Assay for Total Phenolic and Flavonoid Contents

Total phenolics (by Folin–Ciocalteu assay) and flavonoids (by AlCl_3_ assay) were quantified according to the procedures outlined by Slinkard and Singleton (1977). Gallic acid (GA) and rutin (RE) were used as reference standards in the studies, with results expressed as GA equivalents (GAE) and rutin equivalents (RE).

### 2.2 LC–HRMS/MS Analysis

LC–HRMS/MS (Thermo Scientific Vanquish Core HPLC system coupled to the Orbitrap Exploris 120 mass spectrometer, San Jose, CA, USA) was used to determine the metabolic profile of the extract. All parameters and settings of this method are described in detail in our previous work (Stojković et al. 2024). LC/MS data were evaluated using R Studio (version 2023.09.1, build 494) software (Zengin et al. 2020). The phenolic compounds were identified based on their chromatographic behavior and MS and MS^2^ by comparison with standard compounds, when available, and literature data providing a tentative identification. Data acquisition was carried out with Xcalibur data system (Thermo Finnigan, San Jose, CA, USA).

#### 2.2.1 Assays for In Vitro Antioxidant Capacity

In accordance with the methodologies detailed in our prior publication (Grochowski et al. 2017), various antioxidant tests were carried out. The outcomes were represented as milligrams of Trolox equivalents (TE) per gram for the DPPH, ABTS radical scavenging, CUPRAC, and FRAP tests. In millimoles of Trolox equivalents (TE) per gram of extract, the phosphomolybdenum (PBD) test examined antioxidant potential, and in milligrams of disodium edetate equivalents (EDTAE) per gram of extract, the metal chelating activity (MCA) was determined.

#### 2.2.2 Inhibitory Effects Against Some Key Enzymes

In accordance with the established protocols (Grochowski et al. 2017), experiments on enzyme inhibition were performed on the samples. Acarbose equivalents (ACAE) per gram of extract were used to measure the activities that inhibit amylase and glucosidase, while milligrams of galanthamine equivalents (GALAE) per gram of extract was used to examine the inhibition of acetylcholinesterase (AChE) and butyrylcholinesterase (BChE). The amount of tyrosinase inhibition for each gram of extract was measured in milligrams of kojic acid equivalents (KAE).

### 2.3 Evaluation of the Antimicrobial Activity

Antibacterial and antifungal activities were determined using the microdilution method previously described (Zengin et al. 2017). The microorganisms were obtained from the Department of Plant Physiology, Institute for Biological Research “Siniša Stanković,” University of Belgrade, National Institute of the Republic of Serbia.

### 2.4 Molecular Docking Protocol for Ligand–Protein Binding Analysis

The Protein Data Bank (PDB) supplied the proteins and enzymes utilized in this investigation. Table S1 contains the relevant information. Following the retrieval of the structures, the meticulous elimination of co‐crystallized ligands, cofactors, and water molecules was conducted using BIOVIA Discovery Studio Visualizer V4.5. The preparation of the proteins for docking experiments necessitated this step. The ligands were optimized with OpenBabel V3.1.1 subsequent to their acquisition from the PubChem database. In instances where ligands were not identified in PubChem, ChemDraw V1.6.0 was employed to manually delineate their structures prior to optimization (Akyuz Turumtay et al. 2023; Zengin et al. 2024). MGL Tools version 1.5.6 was employed to conduct additional preparations of the protein and enzyme structures, ensuring their suitability for subsequent analysis. The active sites in these proteins were identified using the CavitOmiX V1.0 plugin in PyMOL or literature‐based inhibitor binding sites (Table S1) (Duran et al. 2024; Hetmann et al. 2023; Yagi et al. 2024). Re‐docking was conducted to confirm the accuracy of the initial docking results. The protein was re‐docked with the ligand, and the accuracy of the docking was evaluated by calculating the root mean square deviation (RMSD) values. The following formula was employed to ascertain the RMSD, which is a metric for the mean discrepancy between the atomic coordinates of the reference and target structures:

$$\text{RMSD}=\sqrt{\frac{1}{N}\sum_{i=1}^{N}{\left(r_{i}^{\mathrm{ref}}-r_{i}^{\text{target}}\right)}^{2}}$$

The protein–ligand interactions were further validated using BIOVIA Discovery Studio Visualizer V4.5, specifically focusing on hydrogen bonds and other key interactions. Molecular docking was conducted using AutoDock Vina V1.1.2, with grid boxes configured in accordance with the technique described by Trott and Olson (2010).

### 2.5 MM/PBSA Free Energy Calculations to Assess Ligand Binding Affinity

In this investigation, the gmx_MMPBSA tool (https://valdes‐tresanco‐ms.github.io/gmx_MMPBSA/dev/getting‐started/) was employed to calculate the free energy and evaluate the stability of the compounds. The most stable compounds were selected for investigation based on the outcomes of 10‐ns MD simulations. Subsequently, extended MD simulations (100 ns) were conducted on these selected compounds (Miller III et al. 2012; Valdés‐Tresanco et al. 2021).

### 2.6 MD Simulation Setup for Ligand Stability and Flexibility

Computational MD simulations were initiated using the CHARMM graphical user interface (GUI) platform, accessible at https://charmm‐gui.org/. The simulations were configured using the Solution Builder tool, developed by Jo et al. (2008). The proteins were parameterized using the CHARMM36m force field, as previously described by Yagi et al. (2024) and Maier et al. (2015). The simulated system was confined to a periodic boundary box containing TIP3P water molecules, with a predetermined minimum separation of 10 Å between the protein and the corners of the box. To neutralize the system, counterions were introduced to restore the concentration of NaCl to 0.15 M. The Verlet cutoff approach was used to address electrostatic and van der Waals interactions, while the LINCS algorithm was employed to limit bond lengths. The long‐range electrostatics were computed using the particle mesh Ewald (PME) approach. The steepest descent approach was employed to minimize the energy until the potential energy changes were less than 1000 kJ/mol/nm. To ensure thermodynamic stability, the system was subsequently equilibrated through NVT and NPT phases at 303.3 K. The manufacturing simulation was then conducted using GROMACS 2024.2 for 100 ns (nstep = 50,000,000).

### 2.7 Statistical Analysis

All assays were carried out in triplicate. The results are expressed as mean values and standard deviation (SD). The differences between the tested extracts were analyzed using the student *t*‐test (*p* < 0.05). This treatment was carried out using GraphPad Prism 9.0. program.

## 3 Results and Discussion

### 3.1 Total Phenolic (TPC) and Flavonoids (TFC) Contents

The TPC and TFC of the leaf methanolic extracts of *S. alata* and *S. occidentalis* were determined and the results are presented in Table 1. The TPC and TFC in *S. alata* were 1.5‐ and 1.8‐fold higher than that of *S. occidentalis* . It was observed that the TFC in the two species exceeded their corresponding TPC. Although there is some criticism on the determination of TPC and TFC by spectrophotometric analysis they are still considered rapid, cost‐effective, and acceptable to give a global view of the phenolic content in a sample (Granato et al. 2016; Sánchez‐Rangel et al. 2013; Shraim et al. 2021). The results suggested that flavonoids were the major constituents of the two extracts. Previous studies showed the aqueous and alcoholic extracts of *S. alata* were rich in TPC, in line with the present results (Pamulaparthi et al. 2016; Pukumpuang, Thongwai, and Tragoolpua 2012). Although, *S. occidentalis* displayed relatively lower TPC and TFC than those recorded from *S. alata*, it was noted that these values were higher than those obtained in previous studies (Badock et al. 2024; Ujah, Onyishi, and Onovo 2022). The concentration of phenolics in plants can be influenced by genetic and environmental factors as well as other parameters such as harvest time and extraction techniques (Gramza‐Michałowska et al. 2019).

**TABLE 1:** Total phenolic and flavonoids contents in the leaf methanolic extract of the two investigated Senna species.

Extracts	Total phenolic content (mg GAE/g)	Total flavonoid content (mg RE/g)
*S. alata*	49.95 ± 3.01^a^	82.02 ± 2.57^a^
*S. occidentalis*	33.90 ± 0.89^b^	45.77 ± 0.65^b^

*Note:* Values expressed are means ± SD of three parallel measurements. Different letters indicate significant differences in the tested extracts (*p* < 0.05).

Abbreviations: GAE, Gallic acid equivalent; RE, Rutin equivalent.

### 3.2 Phenolic Profile

Furthermore, the phenolic profile of the leaf methanolic extracts of the two investigated *Senna* species was determined by LC–HRMS/MS and results are presented in Table 2. A total of 75 compounds belonging to dihydroxybenzoic acids, dihydroxycinnamic acids, flavonoid C‐glycosides, flavonoid O‐glycosides, flavonoid aglycones, anthraquinone glycosides, and anthraquinone aglycones were identified. Other phenols like phloridzin, demethylflavasperone 10‐O‐hexoside, 2‐hydroxy‐2,3‐dihydrogenistein, resveratrol, and hedyotisol A were also detected. From these compounds, 55 were identified in *S. alata* and 54 in *S. occidentalis* . Globally, the two species share similar compounds but they have their own specific phytoconstituents which may be reflected in their biological properties. *S. occidentalis* was characterized by the presence of flavonoid C‐glycosides (12 compounds) which were not detected in *S. alata* . The latter accumulated a higher number of dihydroxybenzoic acids (11), dihydroxycinnamic acids (8), and flavonoid O‐glycosides (7) than the former (7, 6, and 5, respectively). The two species had the same anthraquinone aglycones compounds except for two compounds, each found in one species, however, for their anthraquinone glycosides profile, it was observed that sennosides A, B, and C were only identified in *S. alata* . Previous studies isolated/identified flavonoids, anthraquinones, and steroids from the two species, however, the presence of sennosides in *S. alata* were reported for the first time (Fatmawati, Purnomo, and Bakar 2020; Nde et al. 2022). In a previous study by Franca et al. (2021), the presence of some flavonoids including apigenin, kaempferol, and quercetin was reported in the flower extracts of two *Senna* taxa (*Senna splendida* var. *excelsa* and *Senna macranthera*), in accordance with our results. In another study by Guarize et al. (2012), emodin was detected in all extracts of *S. macranthera* leaves. Farag et al. (2015) reported the presence of some anthraquinones including sennosides, rhein, and emodin in the extracts of *S. alexandrina* . However, Navarro et al. (2017) reported that ferulic acid was the major compound in the extract from aerial parts of *Senna reticulata* .

**TABLE 2:** Chemical composition of the leaf methanolic extract of *Senna alata* (SA) and *S. occidentalis* (SO).

No	Compound name	*t*_R_, min	Molecular formula [M−H]^−^	Calculated mass, *m/z*	Exact mass, *m/z*	Δ ppm	MS^2^ fragments (% base peak)	SA	SO	Previously identified in *Senna* (*Cassia*)
Dihydroxybenzoic acids										
1	Dihydroxybenzoyl hexoside	0.82	$C_{13}H_{15}O_{9}^{-}$	315.07216	315.07207	0.27	108.02167 (44), 109.02952 (30), 152.01147 (100), 153.01932 (50), 315.07187 (44)	+	+	NA
2	Dihydroxybenzoyl pentosyl‐hexoside	1.05	$C_{18}H_{23}O_{13}^{-}$	447.11442	447.11364	1.72	108.02176 (4), 109.02959 (11), 152.01152 (100), 153.01892 (5), 447.11407 (82)	+	−	NA
3	Dihydroxybenzoic acid isomer 1	1.15	$C_{7}H_{5}O_{4}^{-}$	153.01933	153.01930	0.24	108.02165 (5), 109.02954 (100), 153.01935 (34)	+	+	Maia et al. (2018)
4	Gallic acid	1.20	$C_{7}H_{5}O_{5}^{-}$	169.01425	169.01418	0.40	125.0245 (29), 151.00371 (100), 169.01427 (90)	+	−	Omer et al. (2022)
5	Hydroxybenzoic acid isomer 1	2.60	$C_{7}H_{5}O_{3}^{-}$	137.02442	137.02438	0.25	93.03457 (100), 137.02449 (27)	+	+	Omer et al. (2022)
6	Dihydroxybenzoic acid isomer 2	2.65	$C_{7}H_{5}O_{4}^{-}$	153.01933	153.01926	0.45	108.02171 (12), 109.02951 (100), 153.01932 (52)	+	+	Maia et al. (2018)
7	Hydroxybenzoic acid isomer 2	4.99	$C_{7}H_{5}O_{3}^{-}$	137.02442	137.02417	1.83	93.03458 (100), 137.02449 (55)	+	−	Omer et al. (2022)
8	Dihydroxybenzoyl‐hydroxybenzoyl hexoside	6.25	$C_{20}H_{19}O_{11}^{-}$	435.09329	435.09274	1.26	101.0244 (25), 109.02940 (20), 137.02426 (40), 152.01143 (100), 153.01918 (45), 281.06656 (24)	+	+	NA
9	Methyl salicylate	6.52	$C_{8}H_{7}O_{3}^{-}$	151.04007	151.04005	0.14	92.0268 (25), 136.01666 (43), 151.04015 (100)	+	+	Zeeshan et al. (2022)
10	Galloyl‐hydroxybenzoyl hexoside	6.93	$C_{20}H_{19}O_{12}^{-}$	451.08820	451.08764	1.23	125.02448 (13), 137.02438 (83), 168.00638 (78), 169.01442 (12), 331.06702 (100)	+	+	NA
11	Hydroxybenzoic acid isomer 3	9.52	$C_{7}H_{5}O_{3}^{-}$	137.02442	137.02444	−0.17	93.03457 (100), 137.02444 (70)	+	−	Omer et al. (2022)
Dihydroxycinnamic acids										
12	*p*‐Coumaroyl hexoside	5.08	$C_{15}H_{17}O_{8}^{-}$	325.09289	325.09286	0.08	119.05025 (56), 145.02945 (14), 163.04010 (100)	+	+	NA
13	*p*‐Coumaroyl pentosyl‐hexoside	5.41	$C_{20}H_{25}O_{12}^{-}$	457.13515	457.13451	1.40	119.05025 (29), 163.04008 (100)	+	−	NA
14	Caffeoylshikimic acid	5.67	$C_{16}H_{15}O_{8}^{-}$	335.07724	335.07714	0.29	135.04515 (50), 161.02454 (23), 179.03493 (100)	+	−	NA
15	*p*‐Coumaric acid	5.80	$C_{9}H_{7}O_{3}^{-}$	163.04007	163.03998	0.53	119.05027 (100), 163.04013 (14)	+	+	Omer et al. (2022)
16	Sinapic acid	6.11	$C_{11}H_{11}O_{5}^{-}$	223.06120	223.06110	0.43	149.02454 (29), 164.04782 (95), 179.07181 (12), 193.01437 (26), 208.03767 (100), 223.06163 (31)	+	+	Chethana et al. (2017)
17	Ferulic acid	6.17	$C_{10}H_{9}O_{4}^{-}$	193.05063	193.05058	0.27	107.0503 (19), 134.03731 (36), 149.06088 (100), 161.04559 (11), 178.02719 (19), 193.05074 (40)	+	+	Omer et al. (2022)
18	Dicaffeoylquinic acid isomer 1	6.36	$C_{25}H_{23}O_{12}^{-}$	515.11950	515.11916	0.66	135.04510 (15), 161.02435 (6), 173.04556 (44), 179.03496 (91), 191.05609 (100), 353.08722 (14)	−	+	Omer et al. (2022)
19	Dicaffeoylquinic acid isomer 2	6.51	$C_{25}H_{23}O_{12}^{-}$	515.11950	515.11904	0.89	135.04509 (11), 173.04546 (100), 179.03487 (77), 191.05605 (31), 353.08698 (17)	−	+	Omer et al. (2022)
20	Sinapoyl‐feruloyl hexoside	6.63	$C_{27}H_{29}O_{13}^{-}$	561.16137	561.16163	−0.47	134.03745 (7), 149.04575 (24), 178.02716 (17), 193.05061 (100), 205.05066 (36), 223.06136 (8)	+	−	NA
21	Caffeic acid	6.63	$C_{9}H_{7}O_{4}^{-}$	179.03498	179.03501	−0.15	135.04556 (4), 179.03508 (100)	+	−	Omer et al. (2022)
Flavonoid C‐glycosides										
22	Luteolin 8‐C‐(2″‐rhamnosyl)‐rhamnoside	5.99	$C_{27}H_{29}O_{14}^{-}$	577.15628	577.15576	0.90	353.06589 (95), 383.07657 (100), 413.08719 (17), 457.11383 (46), 473.10840 (23), 503.11938 (21)	−	+	NA
23	Luteolin 6,8‐di C‐hexoside (Vicenin 2)	5.57	$C_{27}H_{29}O_{15}^{-}$	593.15119	593.15078	0.70	311.05493 (6), 353.06567 (100), 383.07645 (62), 413.08734 (8), 473.10846 (50), 503.11902 (17)	−	+	An et al. (2022)
24	Luteolin 6‐C‐pentoside‐8‐C‐hexoside (isocarlinoside)	5.57	$C_{26}H_{27}O_{15}^{-}$	579.13554	579.13498	0.97	369.06091 (90), 399.07153 (100), 429.08246 (28), 459.09296 (28), 471.09256 (11), 489.10330 (30)	−	+	NA
25	Luteolin 8‐C‐hexoside (orientin)	5.70	$C_{21}H_{19}O_{11}^{-}$	447.09329	447.09247	1.82	297.04059 (10), 299.05588 (8), 327.05072 (100), 357.06088 (74)	−	+	Wei et al. (2024)
26	Apigenin 6‐C‐pentoside‐8‐C‐hexoside (isoschaftoside)	5.77	$C_{26}H_{27}O_{14}^{-}$	563.14063	563.13998	1.15	353.06580 (100), 383.07651 (83), 443.09787 (48), 473.10864 (45), 503.11890 (14)	−	+	NA
27	Isoscoparin 7‐O‐hexoside	5.84	$C_{28}H_{31}O_{16}^{-}$	623.16176	623.16091	1.36	327.05075 (47), 341.06580 (100), 445.07721 (17), 461.10837 (17), 503.12039 (22), 608.13751 (22)	−	+	NA
28	Apigenin 6‐C‐hexoside (isovitexin)	6.09	$C_{21}H_{19}O_{10}^{-}$	431.09837	431.09768	1.60	283.06100 (13), 311.05597 (100), 323.05582 (7), 341.06592 (43)	−	+	Chedjou et al. (2023)
29	Chrysoeriol 6‐C‐hexoside (isoscoparin)	6.18	$C_{22}H_{21}O_{11}^{-}$	461.10894	461.10852	0.89	298.04797 (50), 299.05463 (3), 341.06580 (100), 371.07654 (33)	−	+	NA
30	Malonyl‐(iso)vitexin	6.19	$C_{25}H_{23}O_{14}^{-}$	547.10933	547.10933	0.01	311.05573 (100), 339.08640 (45), 341.06592 (36), 383.07623 (88), 413.08755 (47), 431.09631 (14)	−	+	NA
31	Apigenin 6‐C‐pentoside	6.28	$C_{20}H_{17}O_{9}^{-}$	401.08781	401.08739	1.03	283.06058 (12), 311.05600 (94), 323.05615 (14), 341.06589 (100)	−	+	NA
32	Sinapoyl‐vicenin 2	6.63	$C_{38}H_{39}O_{19}^{-}$	799.20910	799.20855	0.70	353.06570 (100), 383.07639 (76), 413.08670 (14), 455.09750 (38), 473.10846 (29), 575.14008 (32)	−	+	NA
33	Luteolin 8‐C‐(2″‐coumaroyl)‐pentoside	6.95	$C_{29}H_{23}O_{12}^{-}$	563.11950	563.11868	1.45	253.09286 (13), 327.21756 (100), 443.07535 (20), 473.08704 (20)	−	+	NA
Flavonoid O‐glycosides										
34	Kaempferol 3‐O‐(6″‐hexosyl)‐hexoside	5.38	$C_{27}H_{29}O_{16}^{-}$	609.14611	609.14560	0.83	284.03247 (44), 285.04019 (100)	+	+	Abdessadak et al. (2022)
35	Quercetin 3‐O‐(6″‐hexosyl)‐hexoside	5.76	$C_{27}H_{29}O_{17}^{-}$	625.14102	625.14052	0.81	300.02737 (100), 301.03500 (55), 625.14148 (15)	+	+	Kinjo et al. (1994)
36	Isorhamnetin 3‐O‐(6″‐hexosyl)‐hexoside	5.91	$C_{28}H_{31}O_{17}^{-}$	639.15667	639.15649	0.28	299.01953 (77), 300.02676 (22), 314.04315 (100), 315.05032 (17)	+	+	An et al. (2022)
37	Quercetin 3‐O‐hexoside (hyperoside)	6.13	$C_{21}H_{19}O_{12}^{-}$	463.08820	463.08794	0.57	151.00365 (4), 257.04562 (7), 300.02734 (100), 301.03491 (42)	+	+	Omer et al. (2022)
38	Kaempferol 3‐O‐hexoside (astragalin)	6.36	$C_{21}H_{19}O_{11}^{-}$	447.09329	447.09291	0.84	255.02980 (9), 256.03729 (3), 284.03244 (100), 285.04010 (28)	+	−	Omer et al. (2022)
39	Isorhamnetin 3‐O‐hexoside	6.44	$C_{22}H_{21}O_{12}^{-}$	477.10385	477.10351	0.72	299.01953 (13), 300.02734 (100), 314.04318 (8), 315.05069 (21)	+	+	An et al. (2022)
40	Kaempferol 3‐O‐pentoside	6.50	$C_{20}H_{17}O_{10}^{-}$	417.08272	417.08234	0.90	255.03036 (10), 256.03842 (4), 284.03259 (100), 285.04028 (22)	+	−	Yuen et al. (2020)
Flavonoid aglycones										
41	Eriodictyol	7.02	$C_{15}H_{11}O_{6}^{-}$	287.05611	287.05625	−0.47	107.01386 (8), 125.02363 (4), 135.04517 (75), 151.00368 (100)	+	+	Wei et al. (2024)
42	Isorhamnetin	7.06	$C_{16}H_{11}O_{7}^{-}$	315.05103	315.05071	1.01	151.00371 (2), 299.99420 (2), 300.02747 (100), 301.03067 (3), 315.05112 (10)	+	+	Omer et al. (2022)
43	Quercetin	7.07	$C_{15}H_{9}O_{7}^{-}$	301.03538	301.03534	0.13	107.01363 (5), 121.02946 (15), 151.00365 (100), 178.99849 (50), 193.01408 (19), 301.03519 (81)	+	+	Omer et al. (2022)
44	Apigenin	7.45	$C_{15}H_{9}O_{5}^{-}$	269.04555	269.04553	0.05	149.02431 (3), 151.00345 (2), 225.05415 (2), 269.04553 (100)	−	+	Tahir et al. (2023)
45	Naringenin	7.46	$C_{15}H_{11}O_{5}^{-}$	271.06120	271.06110	0.37	107.01379 (11), 119.05021 (38), 151.00363 (100), 165.01956 (3), 177.01933 (13), 271.05914 (70)	+	+	Omer et al. (2022)
46	Chrysoeriol	7.55	$C_{16}H_{11}O_{6}^{-}$	299.05611	299.05583	0.95	256.03726 (2), 284.03244 (100), 299.05606 (33)	+	+	Mahanthesh, Manjappa, Shinde, et al. (2019)
Anthraquinone glycosides										
47	Sennoside B	6.09	$C_{42}H_{37}O_{20}^{-}$	861.18837	861.18752	0.98	224.04787 (46), 386.10001 (100), 448.09454 (12), 449.10117 (12), 610.14899 (16), 655.14270 (6)	+	−	David et al. (2009)
48	8‐Hydroxy‐6‐methoxyrubiadin 3‐O‐(6″‐rhamnosyl)‐hexoside	6.17	$C_{28}H_{31}O_{15}^{-}$	607.16684	607.16789	−1.72	111.00875 (14), 255.06625 (28), 284.03214 (4), 298.04800 (18), 299.05597 (100)	−	+	Tiwari and Singh (1983)
49	Sennoside C	6.28	$C_{42}H_{39}O_{19}^{-}$	847.20910	847.20920	−0.11	224.04779 (27), 386.09995 (100), 479.11273 (14), 640.15851 (7), 641.1662 (14)	+	−	An et al. (2022)
50	Sennoside A	6.35	$C_{42}H_{37}O_{20}^{-}$	861.18837	861.18843	−0.07	224.04774 (42), 386.09991 (100), 448.09482 (9), 449.10202 (11), 610.14795 (11), 655.14478 (6)	+	−	David et al. (2009)
51	Emodin 1‐O‐(6″‐rhamnosyl)‐hexoside	6.40	$C_{27}H_{29}O_{14}^{-}$	577.15628	577.15633	−0.08	269.04538 (100)	−	+	Li et al. (2010)
52	1‐Desmethylaurantioobtusin 2‐O‐hexoside	6.53	$C_{22}H_{21}O_{11}^{-}$	461.10894	461.10856	0.80	283.02454 (44), 284.03217 (13), 298.04797 (50), 299.05573 (30), 446.08478 (100), 461.10831 (80)	+	+	Yang et al. (2017)
53	Emodin 1‐O‐(6″‐hexosyl)‐hexoside	6.84	$C_{27}H_{29}O_{15}^{-}$	593.15119	593.15166	−0.79	268.03751 (27), 269.04532 (100), 311.05582 (4), 593.15094 (35)	+		Wei et al. (2024)
54	1‐Hydroxy‐6,8‐dimethoxy‐2‐methyl‐9,10‐anthracenedione 3‐O‐(6″‐rhamnosyl)‐hexoside	7.09	$C_{29}H_{33}O_{15}^{-}$	621.18249	621.18292	−0.68	269.08188 (26), 312.06400 (18), 313.07166 (100)	−	+	Singh and Singh (1987)
55	Physcion 8‐O‐(6″‐hexosyl)‐hexoside	7.09	$C_{28}H_{31}O_{15}^{-}$	607.16684	607.16749	−1.06	283.06104 (100)	+	+	Wei et al. (2024)
56	Emodin 1‐O‐hexoside	7.22	$C_{21}H_{19}O_{10}^{-}$	431.09837	431.09799	0.88	268.03757 (100), 269.04526 (18)	+	−	Yang et al. (2017)
57	Physcion 8‐O‐hexoside	7.59	$C_{22}H_{21}O_{10}^{-}$	445.11402	445.11343	1.32	240.04344 (9), 283.06113 (100)	−	+	Yang et al. (2017)
58	Emodin 3‐O‐rhamnoside (Frangulin A)	7.73	$C_{21}H_{19}O_{9}^{-}$	415.10346	415.10295	1.23	268.03754 (100), 269.04001 (2)	+	−	Cha et al. (2024)
Anthraquinone aglycones										
59	Emodin	6.48	$C_{15}H_{9}O_{5}^{-}$	269.04555	269.04552	0.09	133.02951 (14), 135.00879 (8), 225.05551 (2), 269.04541 (100)	+	+	Muraoka et al. (2024)
60	Chrysophanol	6.82	$C_{15}H_{9}O_{4}^{-}$	253.05063	253.05065	−0.07	117.03465 (6), 135.00876 (8), 253.05049 (100)	+	+	Gebrehiwot et al. (2024)
61	Physcion	6.91	$C_{16}H_{11}O_{5}^{-}$	283.06120	283.06124	−0.15	268.03760 (100), 283.06113 (40)	+	+	Kinyua et al. (2024)
62	Fistulic acid	7.29	$C_{18}H_{13}O_{8}^{-}$	357.06159	357.06097	1.74	284.03284 (2), 285.03934 (4), 298.04718 (3), 313.07175 (100)	+	−	Agrawal et al. (1972)
63	Flavokermesic acid	7.41	$C_{16}H_{9}O_{7}^{-}$	313.03538	313.03531	0.21	225.05545 (5), 269.04532 (100), 295.02478 (3), 313.03531 (46)	+	+	Alam et al. (2022)
64	8‐Hydroxy‐6‐methoxyrubiadin	7.41	$C_{16}H_{11}O_{6}^{-}$	299.05611	299.05607	0.13	231.06604 (17), 255.06610 (100), 284.03229 (15), 299.05591 (53)	+	+	Tiwari and Singh (1983)
65	Aurantioobtusin	7.49	$C_{17}H_{13}O_{7}^{-}$	329.06668	329.06670	−0.08	285.04031 (97), 299.01959 (62), 311.01974 (35), 314.04309 (100), 329.06821 (28)	+	+	Wei et al. (2024)
66	2‐Hydroxyemodin	7.51	$C_{15}H_{9}O_{6}^{-}$	285.04046	285.04032	0.50	151.00354 (2), 285.04025 (100)	+	+	Zibaee et al. (2023)
67	Lasianthurin B	7.79	$C_{16}H_{11}O_{4}^{-}$	267.06628	267.06626	0.07	117.03464 (17), 252.04271 (89), 267.06601 (100)	+	+	Wei et al. (2024)
68	1‐Desmethylchrysoobtusin	8.45	$C_{18}H_{15}O_{7}^{-}$	343.08233	343.08144	2.58	177.01923 (20), 283.06155 (14), 299.0564 (14), 311.05621 (100)	+	+	Wei et al. (2024)
69	Xanthorin	8.59	$C_{16}H_{11}O_{6}^{-}$	299.05611	299.05625	−0.48	267.02969 (100), 284.03256 (32), 299.05621 (19)		+	Yang et al. (2017)
70	7‐Methoxy obtusifolin	8.60	$C_{17}H_{13}O_{6}^{-}$	313.07176	313.07162	0.45	269.04550 (12), 283.02502 (7), 285.04062 (14), 298.04819 (100), 313.07233 (15)	+	−	Yang et al. (2017)
Other metabolites										
71	Phloridzin	5.44	$C_{21}H_{23}O_{10}^{-}$	435.12967	435.12915	1.19	101.02451 (11), 137.02449 (10), 229.08670 (12), 255.06671 (11), 273.07690 (100)	+	−	Omer et al. (2022)
72	Demethylflavasperone 10‐O‐hexoside	6.46	$C_{21}H_{21}O_{10}^{-}$	433.11402	433.11340	1.44	101.02439 (6), 113.02484 (3), 135.00879 (5), 151.00371 (21), 271.06107 (100)	+	−	Kwon et al. (2021)
73	2‐Hydroxy‐2,3‐dihydrogenistein	6.50	$C_{15}H_{11}O_{6}^{-}$	287.05611	287.05605	0.21	125.02441 (100), 151.00359 (12), 243.06575 (24), 259.06107 (83), 269.04578 (5), 287.05597 (15)	+	+	Lahare et al. (2020)
74	Resveratrol	7.21	$C_{14}H_{11}O_{3}^{-}$	227.07137	227.07135	0.06	143.05016 (6), 157.06589 (3), 159.08145 (5), 183.08205 (6), 185.06082 (22), 227.07130 (100)	+	−	Omer et al. (2022)
75	Hedyotisol A	7.53	$C_{42}H_{49}O_{16}^{-}$	809.30261	809.30231	0.37	150.03221 (25), 165.05563 (100), 195.06613 (34), 417.15536 (3), 565.20837 (7)	+	+	Li et al. (2010)

Abbreviations: −, not detected compounds; +, stands for detected; NA, not available; SA, *Senna alata* ; SO, *Senna occidentalis* ; *t*_R_, retention time (min); Δ ppm, mean mass accuracy.

### 3.3 Antioxidant Activity

Because the assessment of antioxidant parameters involves different effects, including electron donation, hydrogen donation, or metal chelation, no universal method for measuring all antioxidant parameters has been described. With this in mind, most researchers have used different methods to obtain a complete antioxidant picture of a plant extract (Bibi Sadeer et al. 2020). In light of these information, the antioxidant activity of the leaf methanolic extracts of *S. alata* and *S. occidentalis* was determined using six complementary assays and results are presented in Table 3. The antiradical and reducing properties of *S. alata* were significantly higher than *S. occidentalis*. The values recorded in the DPPH, ABTS, CUPRAC, and FRAP assays were respectively 4.3, 3.5, 1.2, and 1.3 times higher than those obtained for *S. occidentalis*. Many studies indicated that the quantity of TPC is proportional to the antioxidant activity (De Marino et al. 2012; Kashyap et al. 2022; Omer et al. 2022). Globally, the findings in the present study align with the TPC and TFC results where *S. alata* exerted significantly (*p* < 0.05) higher content than *S. occidentalis*. On the other hand, *S. occidentalis* exhibited potent chelating capacity, 8.9‐fold higher than *S. alata* . It also revealed slightly higher total antioxidant activity than *S. alata* . Prior studies indicated a limited role of certain polyphenols in chelating metal due to the absence of requisite structural requirements necessary for effective metal chelation (Lu et al. 2007; Yuan, Bone, and Carrington 2005). Previous studies on the antioxidant activity of *S. occidentalis* were mainly evaluated its antiradical activity (Lu et al. 2007; Singh, Jain, and Mishra 2017, 2019; Yuan, Bone, and Carrington 2005). The ethanolic extract of the leaves showed anti‐DPPH activity with an IC_50_ value of 7 μg/mL (Purushotham et al. 2019). Also, the leaves extracts displayed a significant effect in scavenging the hydrogen peroxide (Odeja et al. 2014) while aqueous extract had significant radical scavenging activity (Arya et al. 2011). Panichayupakaranant and Kaewsuwan (2004) showed that the methanol extract of the leaves were more effective in scavenging the DPPH radical (ED_50_ 28.50 μg/mL) than that of the flower (ED_50_ 175.36 μg/mL) and pods (100.18 μg/mL) and from a bioassay‐guided fractionation they found that Kaempferol (ED_50_ 9.99 μg/mL) was more effective than emodin (ED_50_ 578.87 μg/mL). In the present study, beside Kaempferol derivatives and emodin, other compounds known for their antioxidant activity were identified like *p*‐coumaric acid (Kiliç and Yeşiloğlu 2013), sinapic acid (Nićiforović and Abramovič 2014), ferulic acid (Zduńska et al. 2018) flavonoid glycosides (De Marino et al. 2012), and aglycones like eriodictyol (Deng et al. 2020), isorhamnetin (Pengfei et al. 2009), quercetin (Xu et al. 2019), apigenin (Kashyap et al. 2022), naringenin (Cavia‐Saiz et al. 2010), and chrysoeriol (Aboulaghras et al. 2022).

**TABLE 3:** Antioxidant properties of the leaf methanolic extract of the two investigated *Senna* species.

Extracts	DPPH (mg TE/g)	ABTS (mg TE/g)	CUPRAC (mg TE/g)	FRAP (mg TE/g)	Chelating ability (mg EDTAE/g)	Phosphomolybdenum (mmol TE/g)
*S. alata*	58.36 ± 3.86^a^	118.86 ± 2.08^a^	93.85 ± 0.53^a^	50.42 ± 1.51^a^	4.98 ± 0.25^b^	1.39 ± 0.19^b^
*S. occidentalis*	13.46 ± 0.42^b^	34.35 ± 1.87^b^	77.51 ± 3.07^b^	38.34 ± 1.34^b^	44.47 ± 1.95^a^	1.95 ± 0.18^a^

*Note:* Values expressed are means ± SD of three parallel measurements. Different letters indicate significant differences in the tested extracts (*p* < 0.05).

Abbreviations: EDTAE, EDTA equivalent; na, not active; TE, Trolox equivalent.

### 3.4 Enzyme Inhibition Activity

Enzyme inhibition is becoming increasingly important for addressing global health problems. Enzyme inhibition in therapeutic applications is based on the inhibition of key enzymes involved in the pathologies of global health problems such as type II diabetes, Alzheimer's disease, or obesity. By inhibiting important enzymes, the observed symptoms of these diseases can be alleviated. For example, inhibiting cholinesterase can increase acetylcholine levels in the synaptic cleft and improve cognitive function in Alzheimer's patients (de Oliveira et al. 2024). Likewise, inhibiting amylase and glucosidase can delay the rise in blood sugar levels in diabetics (Rangel‐Galván et al. 2024). Based on this information, several compounds have been chemically produced, but most of them have unpleasant side effects such as gastrointestinal problems or toxicity (Huang et al. 2024; Pathak and Kabra 2024). In this sense, we need to replace novel, safe, and effective inhibitors with synthetic ones.

In light of the above points, the enzyme inhibitory activity of the leaf methanolic extracts of *S. alata* and *S. occidentalis* toward the AchE, BchE, Tyr, α‐amylase, and α‐glucosidase enzymes was determined and results are presented in Table 4. Both species displayed comparable effects against the cholinesterase enzymes with remarkable inhibitory activity against the AChE (5.35 and 5.30 mg GALAE/g, *p* ≥ 0.05). Caffeic acid (detected in *S. alata* ) and quercetin (identified in the two species) were shown to possess significant anti‐AchE activity (Szwajgier 2015). Contrary to the present results a previous study showed that the leaf methanolic extract of *S. alata* moderately inhibited the AChE (26.40%) (Azman et al. 2020). Another study showed that the methanol extract from the leaf was not active but the ethyl acetate extract exerted remarkable anti‐AChE activity (IC_50_ 0.08 mg/mL) (Feitosa et al. 2011). Also, the methanolic extract from *S. occidentalis* leaf was shown to effectively inhibit AChE (62.83%) at a concentration of 500 μg/mL (de Almeida et al. 2016).

**TABLE 4:** Enzyme inhibitory properties of the leaf methanolic extract of the two investigated *Senna* species.

Extracts	AChE inhibition (mg GALAE/g)	BChE inhibition (mg GALAE/g)	Tyrosinase inhibition (mg KAE/g)	Amylase inhibition (mmol ACAE/g)	Glucosidase inhibition (mmol ACAE/g)
*S. alata*	5.35 ± 0.04^a^	1.67 ± 0.40^a^	45.07 ± 2.10^a^	0.73 ± 0.04^a^	2.95 ± 0.04^a^
*S. occidentalis*	5.30 ± 0.08^a^	0.64 ± 0.12^b^	23.72 ± 5.28^b^	0.88 ± 0.02^b^	2.40 ± 0.04^b^

*Note:* Values expressed are means ± SD of three parallel measurements. Different letters indicate significant differences in the tested extracts (*p* < 0.05).

Abbreviations: ACAE, acarbose equivalent; GALAE, galatamine equivalent; KAE, Kojic acid equivalent; na, not active.

The capacity of *S. alata* to inhibit Tyr (45.07 mg KAE/g) was significantly (*p* < 0.05) 1.9‐fold higher than that of *S. occidentalis* . These results supported the finding of Saidina et al. (2019) who showed that ethanolic extract of *S. alata* leaf displayed high anti‐Tyr activity (78.75%). However, the whole plant of *S. occidentalis* was found to possess weak anti‐Tyr activity (20%) (Kim et al. 2003). The observed anti‐Tyr activity could be attributed to *p*‐coumaric acid (Boo 2019) and caffeic acid (Crespo et al. 2019).

Moreover, the *Senna* species exhibited a similar effect against the two enzymes linked to diabetes with a higher effect recorded against the α‐glucosidase enzyme (2.95 and 2.40 mmol ACAE/g, *p* ≥ 0.05). The antidiabetic activity of leaf extracts of *S. alata* was previously investigated and results showed that acetone extract significantly inhibited α‐amylase (IC_50_ 6.41 mg/mL), while hexane extract displayed strong inhibition against α‐glucosidase (IC_50_ 0.85 mg/mL) (Varghese, Bose, and Habtemariam 2013). Also, the n‐butanol and ethyl acetate fractions displayed α‐glucosidase inhibitory activity (IC_50_, 25.80 and 2.95 μg/mL, respectively) and kaempferol 3‐O‐gentiobioside was suggested to contribute to this effect (Kazeem, Azeez, and Ashafa 2015). Many in vivo studies demonstrated the antidiabetic activity of *S. occidentalis* and hence the results of the present study via enzyme inhibitory effect suggested that the plant could be a promising source of antidiabetic agents (Nde et al. 2022). Indeed, some compounds were identified in the two species like naringenin (Priscilla et al. 2014), apigenin (Lapchak et al. 2007), and ferulic acid (Li et al. 2022) were proven for their antidiabetic activity. Thus, these two *Senna* species could be a promising source of bioactive molecules with enzyme‐inhibitory properties.

### 3.5 Antibacterial Activity

The antibacterial activity of the leaf methanolic extracts of *S. alata* and *S. occidentalis* was evaluated against 3 Gram‐positive ( *Bacillus cereus* , *Staphylococcus aureus* , and *Listeria monocytogenes* ) and 3 Gram‐negative ( *Escherichia coli* , *Enterobacter cloacae* , and *Salmonella typhimurium* ) bacteria and results are depicted in Table 5. Both species displayed the same antibacterial effect against *B. cereus* , *S. aureus* , and *S. typhimurium* with minimum inhibitory concentration (MIC) ranging between 1.5 and 8 mg/mL and minimum bactericidal concentration (MBC) between 2 and ≥ 8. The best antibacterial activity was recorded against *B. cereus* . However, *S. occidentalis* was more effective against *E. coli* (MIC 3 mg/mL and MBC 4 mg/mL) than *S. alata* while the latter had higher antibacterial activity against *L. monocytogenes* and *E. cloacae* (MIC 6 mg/mL and MBC 8 mg/mL). These results are in line with previous studies that demonstrated the antibacterial potential of *S. alata* and *S. occidentalis* against an array of pathogenic bacteria (Mahanthesh, Manjappa, Sherikar, et al. 2019; Oladeji, Adelowo, and Oluyori 2021). Anthraquinones and flavonoids were suggested to be responsible for this activity. For example, some of the compounds detected in the present study like emodin have been shown to inhibit *E. coli* and *S. aureus* (Khan, Kihara, and Omoloso 2001; Somchit et al. 2003), while, quercetin, naringenin, and glucosides of rhamnetin and chrysoeriol exerted significant antibacterial effects against *Bacillus subtilis* , *Vibrio cholerae* , *Streptococcus* sp., *S. aureus* , and *E. coli* (Tatsimo et al. 2017).

**TABLE 5:** Antibacterial properties of the leaf methanolic extract of the two investigated *Senna* species.

Samples	MIC/MBC (mg/mL)											
	*Bacillus cereus*		*Staphylococcus aureus*		*Listeria monocytogenes*		*Escherichia coli*		*Enterobacter cloacae*		*Salmonella typhimurium*	
	MIC	MBC	MIC	MBC	MIC	MBC	MIC	MBC	MIC	MBC	MIC	MBC
*S. alata*	1.5	2	6	8	6	8	4	8	6	8	8	≥ 8
*S. occidentalis*	1.5	2	6	8	8	≥ 8	3	4	8	≥ 8	8	≥ 8
Streptomycin	0.025	0.050	0.100	0.200	0.150	0.300	0.100	0.200	0.025	0.050	0.100	0.200
Ampicillin	0.100	0.150	0.100	0.150	0.150	0.500	0.150	0.200	0.100	0.150	0.100	0.200

### 3.6 Antifungal Activity

The antifungal activity of the leaf methanolic extracts of *S. alata* and *S. occidentalis* was examined against six fungal species namely, *Aspergillus versicolor*, *Aspergillus flavus*, *Aspergillus niger*, *Penicillium funiculosum*, *Penicillium verrucosum var. cyclopium*, and *Trichoderma viride* and results are presented in Table 6. The two species varied in their capacity to inhibit the tested fungi. *S. alata* displayed a superior effect against 4/6 fungal species. However, the highest antifungal activity was exerted by *S. alata* against *T. viride* (MIC 1 mg/mL and MFC 2 mg/mL) followed by *S. occidentalis* against *A. versicolor* , and *A. flavus* (MIC 2 mg/mL and MFC 4 mg/mL). The other three fungal species were inhibited by the methanolic extract of *S. alata* with MIC 4 mg/mL and MFC 8 mg/mL. A previous study showed that the leaf ethanolic extract of *S. alata* inhibited the growth of *Candida albicans* , *A. niger*, *Penicillium notatum*, *Microsporum canis*, and *Trichophyton mentagrophyte* with MIC values in the range of 3–12 mg/mL (Abubacker, Ramanathan, and Kumar 2008). Also, flower extract and fractions displayed significant antifungal activity against *A. niger* , *Candida utilis* , *Geotrichum candidum*, *Aspergillus brevipes* , and *Penicillium* species with a MIC of 0.312 to 5 mg/mL (Adedayo et al. 1999). The antifungal of *S. occidentalis* leaves against *A. niger* was previously determined by Odeja et al. (2014) but they reported a higher MIC value (25 mg/mL) than that obtained in the present study.

**TABLE 6:** Antifungal properties of the leaf methanolic extract of the two investigated *Senna* species.

Samples	MIC/MFC (mg/mL)											
	*Aspergillus versicolor*		*Aspergillus flavus*		*Aspergillus niger*		*Penicillium funiculosum*		*Trichoderma viride*		*Penicillium verrucosum* var. *cyclopium*	
	MIC	MFC	MIC	MFC	MIC	MFC	MIC	MFC	MIC	MFC	MIC	MFC
*S. alata*	4	8	4	8	4	8	4	8	1	2	4	8
*S. occidentalis*	2	4	2	4	8	≥ 8	8	≥ 8	3	4	8	≥ 8
Ketoconazole	0.200	0.500	0.200	0.500	0.200	0.500	0.200	0.500	1.000	1.500	0.200	0.300
Bifonazole	0.100	0.200	0.150	0.200	0.150	0.200	0.200	0.250	0.150	0.200	0.100	0.200

### 3.7 Evaluating Docking Outcomes: Ligand Binding Energies and Interaction Profiles

The present study employed a holistic assessment approach to evaluate the antimicrobial activities of anthraquinone glycosides identified in *S. alata* and *S. occidentalis* against specific bacterial enzymes, proteins, and standard enzymes. This involved utilizing the coordinates and grid sizes outlined in Table S1. The general identification indicated the presence of a considerable number of compounds, including 2‐hydroxyemodin, 7‐methoxy obtusifolin, aurantioobtusin, emodin 3‐O‐rhamnoside, flavokermesic acid, 1‐desmethylaurantioobtusin 2‐O‐hexoside, sennoside C, 8‐hydroxy 6‐methoxyrubiadin, and sennoside B. The following compounds were selected for comprehensive analysis due to their extensive distribution: chrysophanol, 1‐hydroxy 6,8‐dimethoxy 2‐methyl 9,10‐anthracenedione 3‐O‐hexoside, xanthorin, emodin 1‐O‐(6″‐rhamnosyl)‐hexoside, physcion, emodin, physcion 8‐O‐(6″‐hexosyl)‐hexoside, fistulic acid, and sennoside A. The standard enzymes AChE, BChE, Tyr, amylase, and glucosidase were analyzed in conjunction with the selected proteins for *S. aureus* and *E. coli* , including 30S ribosome S3, dihydropteroate synthase, gyrase B, MurE, and transpeptidase. This was done to investigate the antimicrobial effects of anthraquinone glycosides molecules found in *S. alata* and *S. occidentalis* on these particular bacterial enzymes. Table 7 depicts the compounds with binding energies less than −8 kcal/mol, while Table S2 illustrates those with binding energies greater than −8 kcal/mol. The overall docking data revealed a range of binding energies, from −12.8 to −6.8 kcal/mol (Table S2).

**TABLE 7:** The docking score (kcal/mol) and interacting residues of the enzyme and protein.

Compound	Target	PDB ID	Binding energy	RMSD	Interaction		Binding site
					Type	Number	
Isorhamnetin	Amylase	2qv4	−9.0	0.16	H‐bond	3	Asp A:300, Tyr A:62, Gln A:63
Quercetin 3‐O‐glucoside	Amylase	2qv4	−8.9	1.10	H‐bond	2	Glu A:233, His A:299
3‐O‐Caffeoylquinic acid	Amylase	2qv4	−8.2	0.91	H‐bond	6	Arg A:195, His A:299, Asp A:300, Asp A:197, Glu A:233, Tyr A:62
5‐O‐Caffeoylquinic acid	Amylase	2qv4	−8.3	1.02	H‐bond	3	His A:305, His A:299, Asp A:197
Isorhamnetin 3‐O‐rutinoside	Amylase	2qv4	−9.6	1.08	H‐bond	4	Asp A:197, Glu A:233, Lys A:200, Gln A:63
Rutin	Amylase	2qv4	−9.4	0.86	H‐bond	6	His A:305 (3), Asp A:197, Glu A:233, Lys A:200
Isorhamnetin 3‐O‐glucoside	Amylase	2qv4	−8.4	1.05	H‐bond	2	Glu A:233, Gln A:63
Isorhamnetin	AChE	2y2v	−8.8	1.01	H‐bond	2	Trp A:86, Glu A:202
Quercetin 3‐O‐glucoside	AChE	2y2v	−11.0	1.05	H‐bond	4	Tyr A:133, Glu A:202, Ala A:204, Tyr A:341
3‐O‐Caffeoylquinic acid	AChE	2y2v	−9.3	1.01	H‐bond	2	Glu A:202, Tyr A:124
5‐O‐Caffeoylquinic acid	AChE	2y2v	−9.4	0.11	H‐bond	3	Tyr A:341 (2), Tyr A:124
Isorhamnetin 3‐O‐rutinoside	AChE	2y2v	−10.5	0.96	H‐bond	3	Leu A:76, Ser A:293, Tyr A:341
Rutin	AChE	2y2v	−10.1	1.07	H‐bond	4	Tyr A:337, Asp A:74, Ser A:293, Arg A:296
Isorhamnetin 3‐O‐glucoside	AChE	2y2v	−9.9	1.03	H‐bond	4	Glu A:202, Trp A:86 (2), Gly A:120
Isorhamnetin	BChE	3djy	−9.0	0.30	H‐bond	1	Glu A:197
Quercetin 3‐O‐glucoside	BChE	3djy	−10.1	0.98	H‐bond	8	Gly A:116 (2), Trp A:82, Gly A:115 (2), Tyr A:128, Asp A:70, Tyr A:332
3‐O‐Caffeoylquinic acid	BChE	3djy	−8.3	0.16	H‐bond	4	Pro A:285, Leu A:286, Ala A:199, Gly A:116
5‐O‐Caffeoylquinic acid	BChE	3djy	−8.3	5.32	H‐bond	4	Tyr A:332, Try A:128, Gly A:115, The A:120
Isorhamnetin 3‐O‐rutinoside	BChE	3djy	−11.0	0.80	H‐bond	6	Trp A:82 (2), Gly A:115, Gly A:116, Asp A:70, Pro A:285
Rutin	BChE	3djy	−10.8	0.66	H‐bond	4	Trp A:82, Tyr A:332, Ser A:287, Glu A:197
Isorhamnetin 3‐O‐glucoside	BChE	3djy	−10.1	0.63	H‐bond	4	Trp A:82, His A:438, Ala A:199, Leu A:286
Rutin	Tyr	5m8o	−9.0	0.20	H‐bond	5	Thr A:391, Asp A:212, The A:362, Asn A:378, Ser A:394
Isorhamnetin 3‐O‐rutinoside	*Dihydropteroate synthase*	1ad4	−8.2	0.42	H‐bond	2	Ser A:50, Arg A:219
Rutin	*Dihydropteroate synthase*	1ad4	−8.2	0.57	H‐bond	3	Arg A:66, Gly A:48, His A:55
Rutin	*Gyrase B*	4urn	−8.5	3.53	H‐bond	1	Ala A:122
Rutin	*30S ribosome S3*	5tcu	−8.0	1.07	H‐bond	8	Asp B:17 (2), Arg B:16, The B: 183, Asp B:43, Val B:54, Glu B:48, Arg A:113
Isorhamnetin	*MurE of S. aureus*	4c13	−9.3	8.53	H‐bond	3	Ser A:116, Thr A:115, His A:205
Quercetin 3‐O‐glucoside	*MurEof S. aureus*	4c13	−9.6	5.25	H‐bond	4	Thr A:111, His A:205, Thr A:351, Glu A:460
3‐O‐Caffeoylquinic acid	*MurE of S. aureus*	4c13	−9.8	0.04	H‐bond	11	Thr A:115, Lys A:114 (2), His A:205, Thr A:111, Asn A:301, Ser A:116, His A:353 (2), Gly A:113 (2)
5‐O‐Caffeoylquinic acid	*MurE of S. aureus*	4c13	−9.2	5.87	H‐bond	3	Asp A:350, Thr A:115, Thr A:152
Isorhamnetin 3‐O‐rutinoside	*MurE of S. aureus*	4c13	−10.5	2.22	H‐bond	7	Thr A:115, Gly A:113, Asn A:112, His A:353, Asp A:406, His A:205, Lys A:114
Rutin	*MurE of S. aureus*	4c13	−10.9	6.73	H‐bond	11	Thr A:115, Lys A:114 (2), His A:205, Asn A:407, Asp A:406, Ser A:456, His A:353, Asp A:384, Asp A:204, Asn A:212
Isorhamnetin 3‐O‐glucoside	*MurE of S. aureus*	4c13	−9.1	0.14	H‐bond	8	Ser A:456, Asp A:406, Arg A:383 (2), Ala A:150, Asn A:151, Thr A:115
Quercetin 3‐O‐glucoside	*Transpeptidase of S. aureus*	5tw8	−8.9	1.02	H‐bond	6	Ser A:139, Ser A:75, Ser A:116, Asn A:141, Glu A:114, Glu A:297
3‐O‐Caffeoylquinic acid	*Transpeptidase*	5tw8	−8.4	1.00	H‐bond	5	Ser A:75, Gly A:181, Asp A:264, Arg A:300, Glu A:297
5‐O‐Caffeoylquinic acid	*Transpeptidaseof S. aureus*	5tw8	−8.2	0.23	H‐bond	4	Thr A:260, Ser A:75 Asn A:141, Ser A:262
Isorhamnetin 3‐O‐rutinoside	*Transpeptidase of S. aureus*	5tw8	−9.1	0.11	H‐bond	5	Ser A:139, Ser A:75, Ser A:116, Asp A:264, Glu A:183
Rutin	*Transpeptidaseof S. aureus*	5tw8	−9.3	0.46	H‐bond	4	Thr A:260, Glu A:114, Ser A:75, Glu A:183
Isorhamnetin 3‐O‐glucoside	*Transpeptidaseof S. aureus*	5tw8	−8.7	0.94	H‐bond	8	Glu A:297, Thr A:260, Ser A:75, Ser A:139 (2), Ser A:116, Asn A:141, Glu A:114
Isorhamnetin	*MurE of E. coli*	1e8c	−8.6	0.18	H‐bond	4	Thr B:120, Asp B:356, Asn B:117, His B:359
Quercetin 3‐O‐glucoside	*MurE of E. coli*	1e8c	−9.5	0.04	H‐bond	5	Glu B:468, Lys B:393, Arg B:389, Glu B:182, His 210
3‐O‐Caffeoylquinic acid	*MurE of E. coli*	1e8c	−8.7	0.27	H‐bond	8	Thr B:120, Lys B:119, His B:359, Lys B:393, Asn B:416, Arg B:416, Glu A:468, Gly B:118
5‐O‐Caffeoylquinic acid	*MurE of E. coli*	1e8c	−8.6	0.66	H‐bond	8	Glu B:468, Arg B:416, Asn B:414, Thr B:120, Gly B:118, His B:359, Asp B:209, Lys B:293
Isorhamnetin 3‐O‐rutinoside	*MurE of E. coli*	1e8c	−9.6	0.18	H‐bond	6	Thr B:157, His B:467, Glu B:468, Gly B:464 (2), His B:359
Rutin	*MurE of E. coli*	1e8c	−10.6	1.08	H‐bond	11	Lys B:119 (3), Glu B:155, Arg B:389, Lys B:393, His B:359, Asn B117(2), Gly B:118, Thr B:120
Isorhamnetin 3‐O‐glucoside	*MurE of E. coli*	1e8c	−9.4	0.68	H‐bond	9	Glu B:182, Thr B:116, His B:359 (2), Gly B:464, Asp B:413, Asn B:414, His B:210, Tyr B:470
Quercetin 3‐O‐glucoside	*30S ribosome S3 of E. coli*	4v53	−8.3	0.15	H‐bond	5	Lys B:15 (2), Asp B:182, Asp B:111 (2)
3‐O‐Caffeoylquinic acid	*30S ribosome S3 of E. coli*	4v53	−8.7	0.00	H‐bond	3	Lys B:107, Asn B:7, Asp B:182
5‐O‐Caffeoylquinic acid	*30S ribosome S3 of E. coli*	4v53	−8.6	0.00	H‐bond	3	Asn B:7, Asp B:182, Lys B:15
Isorhamnetin 3‐O‐rutinoside	*30S ribosome S3 of E. coli*	4v53	−8.4	8.84	H‐bond	4	Asn B:18, Asn B:184 (3)
Rutin	*30S ribosome S3 of E. coli*	4v53	−8.7	0.42	H‐bond	4	Asp B:117, Ala B:47, Glu B:109, Gln B:122
Isorhamnetin 3‐O‐glucoside	*30S ribosome S3 of E. coli*	4v53	−8.1	0.99	H‐bond	2	Tyr B:183, Asn B:7
Quercetin 3‐O‐glucoside	*Transpeptidase of E. coli*	6ntw	−8.4	1.03	H‐bond	3	Gly A:582, Gln A:394, Asp A:397
Isorhamnetin 3‐O‐rutinoside	*Transpeptidase of E. coli*	6ntw	−8.7	1.02	H‐bond	8	Gln A:588, Ser A:398, Arg A:590, Ala A:383, Tyr A:384, Asp A:520 (2), Ser A:385
Rutin	*Transpeptidase of E. coli*	6ntw	−8.6	0.88	H‐bond	6	Ala A:383, Arg A:590 (2), Ala A:583, Arg A:522 (2)
Isorhamnetin	*Gyrase B of E. coli*	1kzn	−8.1	1.06	H‐bond	0	
Quercetin 3‐O‐glucoside	*Gyrase B of E. coli*	1kzn	−8.1	0.88	H‐bond	6	Ile A:90, Val A:120, Asp A:73, Glu A:50, Arg A:76 (2)
5‐O‐Caffeoylquinic acid	*Gyrase B of E. coli*	1kzn	−8.1	0.55	H‐bond	5	Ile A:90 Val A:120, Asp A:73 (2), Asn A:46
Isorhamnetin 3‐O‐rutinoside	*Gyrase B of E. coli*	1kzn	−8.2	0.77	H‐bond	4	Glu A:50 (2), Ser A:121, Val A:118
Rutin	*Gyrase B of E. coli*	1kzn	−8.2	0.58	H‐bond	5	Arg A:136, Gly A:77, Asn A:46, Arg A:76, Asp A:73
Isorhamnetin 3‐O‐glucoside	*Gyrase B of E. coli*	1kzn	−8.0	0.91	H‐bond	1	Arg A:76
Quercetin 3‐O‐glucoside	*Dihydropteroate synthase of E. coli*	5v7a	−8.8	0.30	H‐bond	2	Arg A:63, Asp A:96
3‐O‐Caffeoylquinic acid	*Dihydropteroate synthase of E. coli*	5v7a	−8.1	4.57	H‐bond	5	Phe A:188, Thr A:62, Asn A:115, Asp A:185, Arg A:63
5‐O‐Caffeoylquinic acid	*Dihydropteroate synthase of E. coli*	5v7a	−8.2	1.05	H‐bond	2	Asn A:115, Thr A:62
Isorhamnetin 3‐O‐rutinoside	*Dihydropteroate synthase of E. coli*	5v7a	−8.0	6.17	H‐bond	4	Arg A:235 (2), Glu A:60, Asp A:96
Rutin	*Dihydropteroate synthase of E. coli*	5v7a	−8.1	0.84	H‐bond	4	His A:257, Arg A:235, Thr A:62 (2)
Isorhamnetin 3‐O‐glucoside	*Dihydropteroate synthase of E. coli*	5v7a	−8.9	0.00	H‐bond	2	Gly A:189, Arg A:255

**FIGURE 1:**
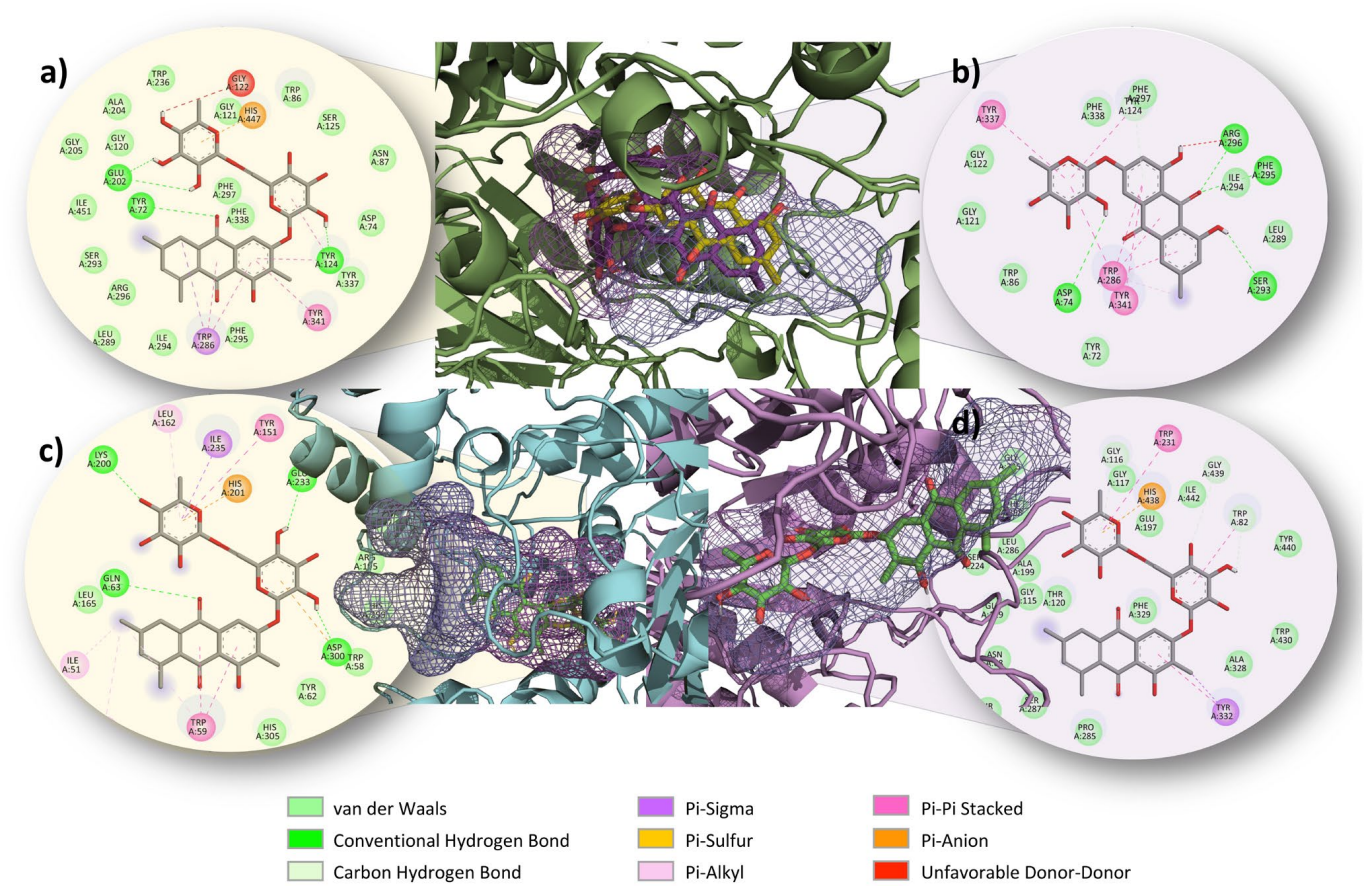
Enzymes and proteins' active sites with compounds showing the best binding energy; (a) Interaction between AChE and 1‐hydroxy 6,8‐dimethoxy 2‐methyl 9,10‐anthracenedione 3‐O‐hexoside. (b) Interaction between AChE and emodin 3‐O‐rhamnoside. (c) Interaction between BChE and 1‐hydroxy 6,8‐dimethoxy 2‐methyl 9,10‐anthracenedione 3‐O‐hexoside. (d) Interaction between amylase and 1‐hydroxy 6,8‐dimethoxy 2‐methyl 9,10‐anthracenedione 3‐O‐hexoside.

**FIGURE 2:**
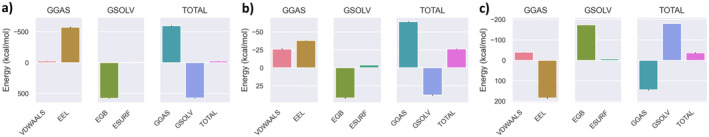
MM/PBSA binding free energy analysis. (a) *E. coli* 30S ribosome S3_sennoside A complex. (b) *S. aureus* gyrase B_chrysophanol complex. (c) *E. coli* MurE_sennoside B complex.

Interactions with binding energies of −8 kcal/mol or lower were found to have RMSD values ranging from 0 to 29.31 Å. An RMSD value greater than 2 Å is indicative of unreliable results, as such values may be indicative of a lack of precision in the data. The selection of these complexes was based on key parameters such as binding energies, hydrogen bond counts, and RMSD values. The lowest binding energies were observed with AChE and 1‐hydroxy 6,8‐dimethoxy 2‐methyl 9,10‐anthracenedione 3‐O‐hexoside (−12.8 kcal/mol; RMSD value: 0.53) (Figure 1a). Similarly, AChE and emodin 3‐O‐rhamnoside (−11.7 kcal/mol; RMSD value: 0.85) (Figure 1b) showed low binding energies, as did Physcion 8‐O‐hexoside, which exhibited strong interactions with AChE, showing a binding energy of −10.2 kcal/mol with an RMSD value of 0.04 Å, forming six hydrogen bonds with residues Tyr A:72, Tyr A:124, Phe A:295, Ser A:293 (2), and Gln A:291. BChE and 1‐hydroxy 6,8‐dimethoxy 2‐methyl 9,10‐anthracenedione 3‐O‐hexoside (−11.4 kcal/mol; RMSD value: 1.05) (Figure 2c) showed strong interactions. Similarly, sennoside C showed significant interactions with BChE, with a binding energy of −10.3 kcal/mol and an RMSD value of 0.04 Å, forming five hydrogen bonds with residues Ser A:79, His A:438 (2), Trp A:82, and Ser A:287. Moreover, amylase and 1‐hydroxy 6,8‐dimethoxy 2‐methyl 9,10‐anthracenedione 3‐O‐hexoside (−11.2 kcal/mol; RMSD value: 0.80) (Figure 2d) also showed notable interactions. Sennoside C and sennoside A showed −10.0 and −9.2 kcal/mol, respectively, upon binding with amylase. Sennoside C has shown less fluctuation of 0.68 Å compared to sennoside A, which has an RMSD value of 0.91 Å. Sennoside C forms six hydrogen bonds interacting with residues Glu A:233, Asp A:300 (2), and His A:305 (3). In contrast, sennoside A formed nine hydrogen bonds with residues Lys A:200, Glu A:240, Ile A:148, Tyr A:151, Asp A:300 (2), His A:299, Trp A:59, and His A:201. Additionally, sennoside B and 1‐hydroxy‐6,8‐dimethoxy‐2‐methyl‐9,10‐anthracenedione 3‐O‐hexoside showed remarkable interactions with glucosidase. Sennoside B displayed a binding energy of −9.5 kcal/mol with an RMSD value of 0.24 Å, forming six hydrogen bonds with residues Asp A:232, Lys A:506, Phe A:476, Asp A:357, and Asp A:469 (2). In contrast, 1‐hydroxy‐6,8‐dimethoxy‐2‐methyl‐9,10‐anthracenedione 3‐O‐hexoside exhibited strong interactions with glucosidase, showing a binding energy of −10.1 kcal/mol with an RMSD value of 0.25 Å, forming six hydrogen bonds with residues His A:626, Asp A:357, Arg A:552 (2), Asp A:568, and Phe A:476.

Sennoside B showed significant interactions with MurE from *E. coli* . Sennoside B showed a binding energy of −9.7 kcal/mol with an RMSD value of 0.40 Å, forming four hydrogen bonds with residues Asn B:156, Glu B:155, Arg B:29, and Asn B:90. Sennoside C, emodin 1‐O‐hexoside, and physcion 8‐O‐hexoside exhibited notable interactions with transpeptidases of *S. aureus* and *E. coli* . Sennoside C, when docked with the transpeptidase of *S. aureus* , showed a binding energy of −9.5 kcal/mol with an RMSD value of 0.65 Å, forming seven hydrogen bonds with residues Asn A:141, Ser A:262, Ser A:75, Ser A:116, Ser A:139, Glu A:297, and Tyr A:291. Emodin 1‐O‐hexoside demonstrated a binding energy of −10.0 kcal/mol with an RMSD value of 0.96 Å, forming 10 hydrogen bonds with residues Thr A:180, Ala A:182, Ser A:75 (3), Ser A:116, Ser A:139 (2), and Thr A:260. Physcion 8‐O‐hexoside exhibited a binding energy of −9.4 kcal/mol with an RMSD value of 0.51 Å, forming six hydrogen bonds with residues Thr A:180, Ser A:75, Asn A:141, Ser A:116, and Ser A:139 (2). 30S ribosome S3 of *E. coli* with sennoside A showed a binding energy of −9.0 kcal/mol with an RMSD value of 0.62 Å, forming five hydrogen bonds with residues Asn B:184 (2), Asn B:18, Asp B:182, and Ser B:19. Transpeptidase of *E. coli* , sennoside C displayed a binding energy of −9.3 kcal/mol with an RMSD value of 0.54 Å, forming four hydrogen bonds.

Sennoside C, chrysophanol, 1‐hydroxy 6,8‐dimethoxy 2‐methyl 9,10‐anthracenedione 3‐O‐hexoside, xanthorin, emodin 1‐O‐hexoside, and sennoside A with the MurE enzyme of *S. aureus* . Sennoside C exhibited a binding energy of −9.7 kcal/mol with an RMSD value of 0.94 Å, forming nine hydrogen bonds with residues Arg A:187, Asn A:151, Thr A:28, Thr A:27, Arg A:31, Thr A:46, Thr A:45, and Val A:47 (2). Chrysophanol displayed a binding energy of −10.5 kcal/mol with an RMSD of 0.88 Å, forming four hydrogen bonds with residues Ser A:116 (3) and Asn A:301. 1‐Hydroxy 6,8‐dimethoxy 2‐methyl 9,10‐anthracenedione 3‐O‐hexoside showed a binding energy of −9.6 kcal/mol with an RMSD of 0.39 Å, forming five hydrogen bonds with residues Arg A:383, Ala A:150, Arg A:187 (2), and Thr A:137. Xanthorin exhibited a binding energy of −10.2 kcal/mol with an RMSD of 0.91 Å, forming four hydrogen bonds with Thr A:115, Ser A:116 (2), and Asn A:301. Emodin 1‐O‐hexoside had a binding energy of −8.9 kcal/mol with an RMSD value of 0.76 Å, forming 10 hydrogen bonds with residues Thr A:152 (2), Arg A:383 (2), His A:205, Thr A:351 (2), Glu A:460, and Thr A:462. Finally, sennoside A demonstrated a binding energy of −10.2 kcal/mol with an RMSD of 0.43 Å, forming 10 hydrogen bonds with Ser A:30 (2), Thr A:28, Arg A:187 (3), Asn A:151 (3), and Arg A:31. 1‐Hydroxy 6,8‐dimethoxy 2‐methyl 9,10‐anthracenedione 3‐O‐hexoside exhibited significant interactions with the 30S ribosome S3 of *S. aureus* . This compound displayed a binding energy of −9.3 kcal/mol with an RMSD value of 0.12 Å, forming five hydrogen bonds with residues Ala B:20, Glu B:19, Arg A:39 (2), and Asp B:17. Sennoside C exhibited notable interactions with dihydropteroate synthase of *E. coli* . Sennoside C showed a binding energy of −8.4 kcal/mol with an RMSD value of 0.21 Å, forming five hydrogen bonds with residues Gln A:149, Gly A:189, Thr A:62, Arg A:63, and His A:257.

These data indicate that sennoside C, sennoside B, and 1‐hydroxy‐6,8‐dimethoxy‐2‐methyl‐9,10‐anthracenedione 3‐O‐hexoside can be used as potential inhibitors against mainly AChE, BChE, amylase, and glucosidase enzymes. In addition, low RMSD values of these compounds further establish their structural stability and the reliability of their interactions with the enzymes. Further studies in the future need to focus on substantiating the in vitro and in vivo inhibitory mechanisms of these compounds and their pharmacological applications. The present study also showed that sennoside B, sennoside C, emodin 1‐O‐hexoside, physcion 8‐O‐hexoside, chrysophanol, and other anthraquinone derivatives exhibited effective antibacterial activities against some pathogenic bacteria such as *E. coli* and *S. aureus* . They had high binding energies with vital bacterial targets, especially the MurE enzyme, transpeptidases, and 30S ribosome S3, suggesting that they could inhibit bacterial cell wall synthesis and protein production. High binding affinities along with multiple hydrogen bonds with these bacterial targets mark their potential as new antibacterial drug candidates.

### 3.8 Binding Free Energy Analysis: MM/PBSA Results and Implications for Ligand Efficacy

This study entailed the calculation of the energy components of several protein–complex interactions and a comprehensive analysis of their implications on binding stability. A comprehensive examination was conducted on the energy values pertaining to the Van der Waals interaction (VDWAALS), electrostatic energy (EEL), polar solvation (EGB), surface tension (ESURF), gas phase energy (GGAS), solvation energy (GSOLV), and total energy (TOTAL) for each complex. *E. coli* and *S. aureus* activities of anthraquinone glycosides compounds derived from *S. alata* and *S. occidentalis* were analyzed using MD simulations and MM/PBSA binding free energy calculations. Based on criteria such as low RMSD, high binding energy, and the number of hydrogen bonds, nine complexes were selected for further analysis. These selected complexes include: *E. coli* 30S ribosome S3_sennoside A, *S. aureus* 30S ribosome S3_1‐hydroxy 6,8‐dimethoxy 2‐methyl 9,10‐anthracenedione 3‐O‐hexoside, *E. coli* dihydropteroate synthase_sennoside C, *E. coli* gyrase B_senoside A, *S. aureus* gyrase B_chrysophanol, *S. aureus* MurE_chrysophanol, *E. coli* MurE_sennoside B, *S. aureus* transpeptidase_emodin 1‐O‐hexoside, and *S. aureus* dihydropteroate synthase_sennoside B (Table S3).

Based on the data presented in Figure 2, three complexes emerge as particularly stable in terms of binding. The complexes identified as exhibiting the most promising binding stability are *E. coli* 30S ribosome S3_sennoside A, *E. coli* gyrase B_senoside A, and *E. coli* MurE_sennoside B. These complexes are highlighted in red in the table. The electrostatic energy value of −572.36 kcal/mol and the polar solvation energy of 577.86 kcal/mol in the *E. coli* 30S ribosome S3_sennoside A complex are particularly noteworthy, and the total energy value of −24.93 kcal/mol indicates strong binding stability. In terms of binding affinity, two other complexes merit particular mention: *S. aureus* gyrase B_chrysophanol (−26.3 kcal/mol) and *S. aureus* transpeptidase_emodin 1‐O‐hexoside (−14.28 kcal/mol). In comparison to the other complexes, the *S. aureus* dihydropteroate synthase_dennoside B complex exhibits diminished binding stability, with a total energy of −9.88 kcal/mol (Table S3). Overall, these findings demonstrate that sennoside A, sennoside B, and chrysophanol derivatives possess robust binding affinities to protein targets, suggesting their potential as inhibitor candidates and a significant contribution to drug development research.

### 3.9 Dynamic Stability and Structural Flexibility: Insights From MD Simulations

**FIGURE 3:**
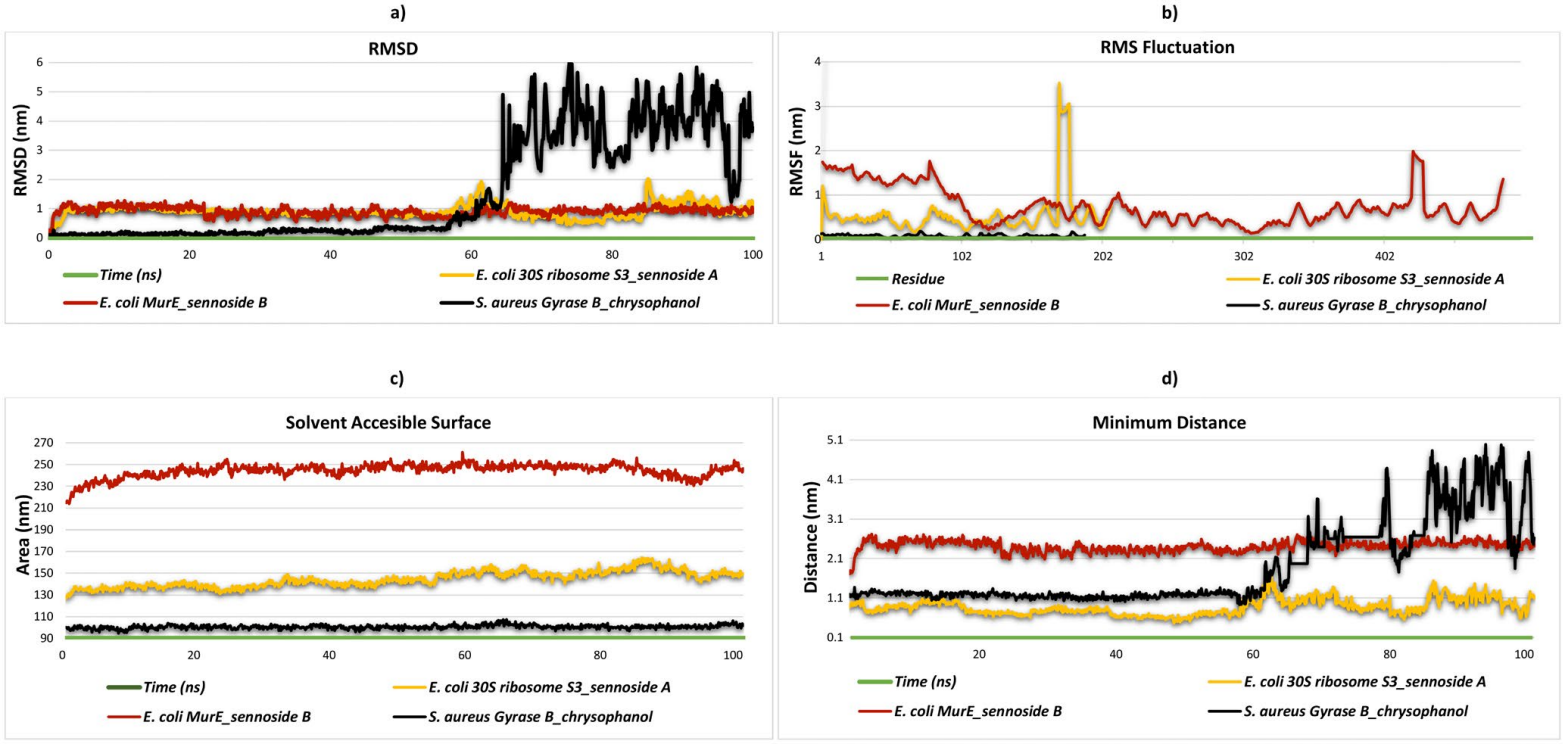
Presentation of molecular dynamics simulations in graphical form. (a) RMSD of *E. coli* 30S ribosome S3_sennoside A complex, *E. coli* MurE_sennoside B complex, and *S. aureus* gyrase B_chrysophanol complex. (b) RMSF of *E. coli* 30S ribosome S3_sennoside A complex, *E. coli* MurE_sennoside B complex, and *S. aureus* gyrase B_chrysophanol complex. (c) Minimum distance of *E. coli* 30S ribosome S3_sennoside A complex, *E. coli* MurE_sennoside B complex, and *S. aureus* gyrase B_chrysophanol complex. (c) Solvent accessibility of *E. coli* 30S ribosome S3_sennoside A complex, *E. coli* MurE_sennoside B complex, and *S. aureus* gyrase B_chrysophanol complex. (d) Radius of gyration of *E. coli* 30S ribosome S3_sennoside A complex, *E. coli* MurE_sennoside B complex, and *S. aureus* gyrase B_chrysophanol complex.

The objective of this study is to identify potential therapeutic agents through a comprehensive analysis of the molecular interactions between ligands and proteins to identify binding sites. Nine potential ligands were subjected to a detailed examination to assess their biological potency and ability to interact with proteins. The ligands were selected based on their MM/PBSA binding and molecular docking scores. *S. aureus* gyrase B_chrysophanol complex, *E. coli* MurE_sennoside B complex, and *E. coli* 30S ribosome S3_sennoside A complex were selected. These complexes, which are characterized by selectivity and stability of interactions with the target proteins, were found to be critical for MD simulations. The RMSD plot illustrates how the RMSD of each ligand varies over time. While the *S. aureus* gyrase B_chrysophanol complex shows a larger and more variable RMSD, sennoside A with *E. coli* 30S ribosome S3 and sennoside B with *E. coli* MurE follow a relatively lower and consistent pattern (Figure 3a). MD simulations serve to elucidate the regions of flexibility and stability in binding by illuminating the dynamic interplay between residues and ligands in protein–ligand complexes. Sennoside A forms hydrogen bonds with residues Asn b:184 (double bond), Asn b:18, Asp b:182, and Ser b:19 to interact with the *E. coli* 30S ribosome S3. As illustrated by the RMSF graph, this area displays remarkable flexibility. The contact remains stable due to the presence of hydrogen bonds, although there is a notable increase in mobility at residue 200. The residues responsible for the binding of sorboside B to *E. coli* MurE are Asn b:156, Glu b:155, Arg b:29, and Asn b:90. In contrast, residue 200 exhibits higher RMSF values, indicating greater flexibility. Despite the region's dynamic character, strong hydrogen bonds contribute to the stabilization of this interaction. In contrast, chrysophanol binds to *S. aureus* gyrase B through residues Arg a:79, Gly a:80 (double bond), and Thr a:168, and the RMSF graph indicates low flexibility, suggesting a highly stable interaction in this region (Figure 3b).

The solvent‐accessible surface area (SASA) analysis reveals distinct solvent exposure profiles for each protein–ligand complex. The highest SASA values are observed in *E. coli* MurE_sennoside B, indicating that the ligand binding site remains highly accessible despite considerable solvent exposure. In contrast, *E. coli* 30S ribosome S3_sennoside A exhibits intermediate SASA values with a modest increasing trend, indicating a dynamic binding area and moderate solvent exposure. Conversely, *S. aureus* gyrase B_chrysophanol exhibits the lowest SASA values, indicating a more compact or buried interaction with restricted solvent accessibility. The binding affinity, stability, and general dynamics of each complex in the solvent environment may be influenced by these alterations in solvent exposure (Figure 3c). Moreover, the data obtained from MD simulations offers substantial new insights into the minimal distances between protein and ligand complexes. The lowest recorded distances in the *E. coli* 30S ribosome S3_sennoside A complex data ranged from 0.70 to 1.6 nm, with an average value of 0.87 nm. The minimum distances observed for the *E. coli* MurE_sennoside B complex ranged from 1.7 to 2.7 nm, with an average value of 1.6 nm. The minimum distances for the *S. aureus* gyrase B_chrysophanol complex ranged from 0.96 to 4.9 nm, with an average of 2.1 nm. These findings indicate that the *E. coli* 30S ribosome S3_sennoside A and *E. coli* MurE_sennoside B complexes demonstrate more stable interactions with lower and more consistent minimum distances, while the *S. aureus* gyrase B_chrysophanol complex exhibits more variable and higher minimum distances (Figure 3d).

**FIGURE 4:**
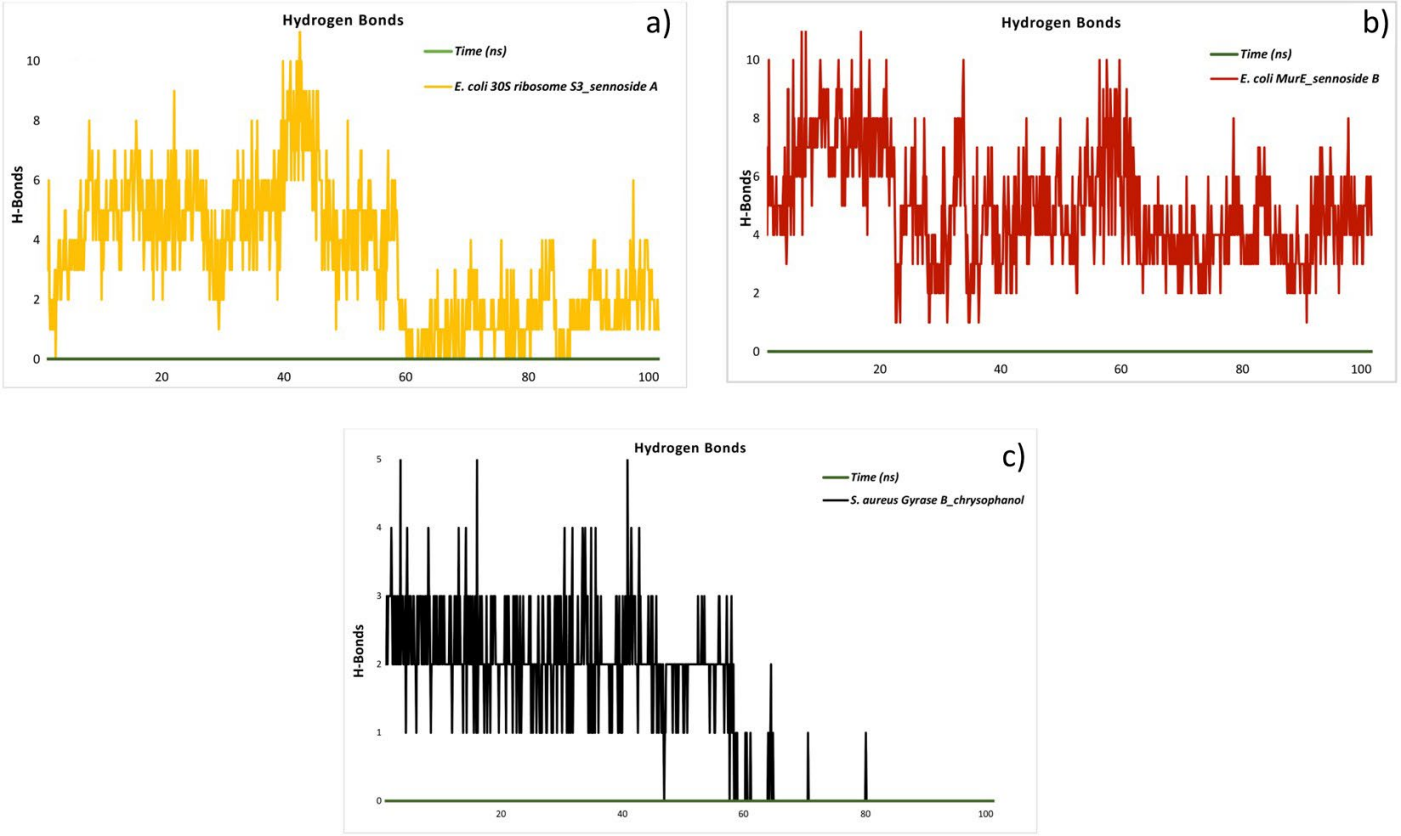
Hydrogen bonds in *E. coli* 30S ribosome S3_sennoside A complex (a). Hydrogen bonds in *E. coli* MurE_sennoside B complex (b). Hydrogen bonds in *S. aureus* gyrase B_chrysophanol complex (c).

MD simulations provide substantial insights into the minimum distances between proteins and ligands in complexes, as well as the kinetics of hydrogen bonding (Figure 4). The results for the *E. coli* 30S ribosome S3_sennoside A complex indicate that hydrogen bonds can form in a range of 0–11, with an average of three and sporadic variations. The number of hydrogen bonds remains stable at four up until 60 ns, after which it drops to one between 60 and 90 ns. Following a period of 90 ns, the number of hydrogen bonds stabilizes at two once more, indicating that the binding interactions underwent dynamic changes during the course of the experiment. The *E. coli* MurE_sennoside B complex reaches a maximum of 11 hydrogen bonds and generally remains stable between four and five hydrogen bonds. The hydrogen bond profile of the *S. aureus* gyrase B_chrysophanol complex indicates that during the initial phase of the simulation, the number of hydrogen bonds remains relatively consistent, oscillating between two and three. Nevertheless, the complex is observed to cease forming hydrogen bonds after 60 ns, indicating that as the simulation progresses, hydrogen bond interactions are lost. This suggests that the binding connection will eventually become weaker or even break.

These results illustrate the dynamic nature of protein–ligand interactions and the crucial role of hydrogen bonding in maintaining binding stability over time. The *E. coli* 30S ribosome S3_sennoside A complex exhibited variable yet recoverable hydrogen bond connections, whereas the *S. aureus* gyrase B_chrysophanol complex demonstrated a loss of bonding capacity over the course of the simulation, indicating a potential decline in binding affinity. The *E. coli* MurE_sennoside B complex exhibited a higher and more consistent number of hydrogen bonds, indicating a more stable and potentially stronger interaction. In light of these findings, the utility of MD simulations in drug development is underscored by their ability to illustrate these subtle yet significant alterations in solvent accessibility and hydrogen bonding. These insights can inform the identification and optimization of lead compounds, facilitating the prediction of their therapeutic potential by elucidating the interaction dynamics at the molecular level.

## 4 Conclusion

The present study entailed the first comprehensive examination of the chemical composition of *S. alata* and *S. occidentalis* . Results indicated that the leaves of the two species were rich in phenolics with super TPC and TFC recorded in *S. alata* . *S. occidentalis* was characterized by the presence of flavonoid C‐glycosides while *S. alata* accumulated a higher number of dihydroxybenzoic acids, dihydroxycinnamic acids, and flavonoid O‐glycosides. Also, Sennosides A, B, and C were only detected in *S. alata*. Both species revealed significant antioxidant, enzyme inhibition, antibacterial, and antifungal activities with *S. alata* recording higher effect in most assays. The *Senna* species in question has been proven to have antibacterial activity, particularly due to the presence of specific molecules, including sennoside A and sennoside B. In conclusion, the two species displayed multidirectional biological properties, and they could be a promising source of raw materials for health‐promoting applications. Further studies including the isolation of bioactive compounds and identification of their pharmacokinetic properties are recommended.

## Author Contributions

**Sakina Yagi:** conceptualization (equal), data curation (equal), formal analysis (equal), writing – original draft (equal), writing – review and editing (equal). **Mehmet Veysi Cetiz:** conceptualization (equal), data curation (equal), visualization (equal), writing – original draft (equal), writing – review and editing (equal). **Gokhan Zengin:** investigation (equal), methodology (equal), writing – original draft (equal), writing – review and editing (equal). **Kassim Bakar:** investigation (equal), methodology (equal), resources (equal), writing – original draft (equal), writing – review and editing (equal). **Azali Ahamada Himidi:** investigation (equal), methodology (equal), resources (equal), writing – review and editing (equal). **Andilyat Mohamed:** conceptualization (equal), investigation (equal), methodology (equal), validation (equal), writing – original draft (equal), writing – review and editing (equal). **Marijana Skorić:** investigation (equal), methodology (equal), writing – original draft (equal), writing – review and editing (equal). **Jasmina Glamočlija:** investigation (equal), methodology (equal), writing – original draft (equal), writing – review and editing (equal). **Uroš Gašić:** investigation (equal), methodology (equal), writing – original draft (equal), writing – review and editing (equal).

## Ethics Statement

The authors have nothing to report.

## Consent

Written informed consent was obtained from all study participants.

## Conflicts of Interest

The authors declare no conflicts of interest.

## Supporting information


Table S1.
Table S2.
Table S3.

## Data Availability

The data that support the findings of this study are available on request from the corresponding author.
