# Supplementary material for: Novel Natural Candidates for Replacing Synthetic Additives in Nutraceutical and Pharmaceutical Areas: Two Senna Species (S. alata (L.) Roxb. and S. occidentalis (L.) Link)
Source: Food Sci Nutr. 2024 Dec 22;13(1):e4705. doi: 10.1002/fsn3.4705 (PMC11717048; doi:10.1002/fsn3.4705)
Supplement: Supplementary file 1 — Table S1. Table S2. Table S3. [file FSN3-13-e4705-s001.docx]

**Supplementary Tables**

**Table S1.** Relevant protein and enzyme target coordinates of the docking box.

| **Group** | **Target** | **PDB ID** | **Grid size X, Y, Z** | **X, Y, Z dimensions** | **Reference** |
| --- | --- | --- | --- | --- | --- |
| **Enzyme** | AChE | 2y2v | 22 Å X 30 Å X 40 Å | 31.062, 20.311, 11.947 | (Yagi et al., 2024} |
|  | BChE | 3djy | 30 Å X 30 Å X 30 Å | 44.794, -19.63, -25.227 | (Duran et al., 2024) |
|  | Tyr | 5m8o | 26 Å X 26 Å X 28 Å | -13.194, 5.341, -26.28 | (Yagi et al., 2024 |
|  | Amylase | 2qv4 | 28 Å 28 Å X 24 Å | 14.188, 48.964, 22.886 | (Yagi et al., 2024) |
|  | Glucosidase | 3w37 | 42 Å 52 Å X 54 Å | 3.091, −8.008, −4.08 | (Duran et al., 2024) |
| ***S. aureus*** | 30S ribosome S3 | 5tcu | 76 Å x 76 Å x 76 Å | 99.46, 230.082, 201.387 | (Saqallah et al., 2022) |
|  | Dihydropteroate synthase | 1ad4 | 60 Å x 60 Å x 60 Å | 32.46, 6.683, 42,972 | (Saqallah et al., 2022;Hetmann et al., 2023) |
|  | Gyrase B | 4urn | 60 Å x 60 Å x 40 Å | -31.684, -5.252, 1.572 | (Saqallah et al., 2022; Hetmann et al., 2023) |
|  | MurE | 4c13 | 60 Å x 60 Å x 60 Å | -23.122, 2.508, 9.873 | (Saqallah et al., 2022; Hetmann et al., 2023) |
|  | Transpeptidase | 5tw8 | 60 Å x 60 Å x 60 Å | 21.390, -62.210, 39.196 | (Saqallah et al., 2022) |
| ***E. coli*** | 30S ribosome S3 | 4v53 | 88 Å x 88 Å x 88 Å | 130.966, 32.099, 0.385 | (Saqallah et al., 2022; Hetmann et al., 2023) |
|  | Dihydropteroate synthase | 5v7a | 60 Å x 60 Å x 60 Å | -17.836, -17.836, 103.740 | (Saqallah et al., 2022) |
|  | Gyrase B | 1kzn | 60 Å x 60 Å x 60 Å | 12.467, 27.336, 44.916 | (Saqallah et al., 2022; Hetmann et al., 2023) |
|  | MurE | 1e8c | 70 Å x 60 Å x 70 Å | 45.098, 37.112, 76.674 | (Saqallah et al., 2022; Hetmann et al., 2023) |
|  | Transpeptidase | 6ntw | 60 Å x 60 Å x 60 Å | 16.929, -32.370, 42.151 | (Saqallah et al., 2022; Hetmann et al., 2023) |

**Table S2** Relevant protein and enzyme result of the docking scores

| **Group** | **Compound** | **PDB ID** | **Binding energy** | **PDB ID** | **Binding energy** | **PDB ID** | **Binding energy** | **PDB ID** | **Binding energy** | **PDB ID** | **Binding energy** |
| --- | --- | --- | --- | --- | --- | --- | --- | --- | --- | --- | --- |
| **Enzyme** | 2-Hydroxyemodin | 2qv4 | -8.4 | 3w37 | -7.5 | 2y2v | -9.5 | 3djy | -8.8 | 5m8o | -7.0 |
|  | 7-Methoxy obtusifolin | 2qv4 | -8.0 | 3w37 | -7.4 | 2y2v | -9.4 | 3djy | -8.3 | 5m8o | -7.0 |
|  | Aurantioobtusin | 2qv4 | -8.4 | 3w37 | -7.0 | 2y2v | -9.1 | 3djy | -8.2 | 5m8o | -6.9 |
|  | Emodin 3-O-rhamnoside | 2qv4 | -11.0 | 3w37 | -8.9 | 2y2v | -11.7 | 3djy | -10.4 | 5m8o | -8.1 |
|  | Flavokermesic acid | 2qv4 | -8.4 | 3w37 | -7.4 | 2y2v | -10.9 | 3djy | -8.8 | 5m8o | -7.1 |
|  | 1-Desmethylaurantioobtusin 2-O-hexoside | 2qv4 | -8.6 | 3w37 | -7.3 | 2y2v | -9.9 | 3djy | -9.0 | 5m8o | -6.8 |
|  | Sennoside C | 2qv4 | -10.0 | 3w37 | -9.9 | 2y2v | -8.2 | 3djy | -10.3 | 5m8o | -8.7 |
|  | 8-Hydroxy 6-methoxyrubiadin | 2qv4 | -8.1 | 3w37 | -7.2 | 2y2v | -9.3 | 3djy | -8.6 | 5m8o | -7.1 |
|  | Sennoside B | 2qv4 | -9.4 | 3w37 | -9.5 | 2y2v | -8.3 | 3djy | -9.5 | 5m8o | -8.2 |
|  | Chrysophanol | 2qv4 | -8.3 | 3w37 | -7.2 | 2y2v | -9.7 | 3djy | -9.1 | 5m8o | -6.8 |
|  | 1-Hydroxy 6,8-dimethoxy 2-methyl 9,10-anthracenedione 3-O-hexoside | 2qv4 | -11.2 | 3w37 | -10.1 | 2y2v | -12.8 | 3djy | -11.4 | 5m8o | -8.5 |
|  | Xanthorin | 2qv4 | -8.4 | 3w37 | -7.3 | 2y2v | -9.5 | 3djy | -8.4 | 5m8o | -6.9 |
|  | Emodin 1-O-(6"-rhamnosyl)-hexoside | 2qv4 | -9.4 | 3w37 | -8.4 | 2y2v | -9.9 | 3djy | -10.5 | 5m8o | -7.8 |
|  | Physcion | 2qv4 | -8.3 | 3w37 | -7.4 | 2y2v | -9.5 | 3djy | -8.5 | 5m8o | -7.2 |
|  | Emodin | 2qv4 | -8.5 | 3w37 | -7.5 | 2y2v | -9.5 | 3djy | -9.1 | 5m8o | -6.9 |
|  | Physcion 8-O-(6"-hexosyl)-hexoside | 2qv4 | -9.4 | 3w37 | -8.2 | 2y2v | -10.2 | 3djy | -10.0 | 5m8o | -7.4 |
|  | Fistulic acid | 2qv4 | -8.7 | 3w37 | -7.5 | 2y2v | -8.9 | 3djy | -8.2 | 5m8o | -6.8 |
|  | Sennoside A | 2qv4 | -9.2 | 3w37 | -9.3 | 2y2v | -8.1 | 3djy | -9.7 | 5m8o | -8.4 |
| ***S. aureus*** | 2-Hydroxyemodin | 1ad4 | -6.9 | 4urn | -8.1 | 5tcu | -7.3 | 4C13 | -10.2 | 5TW8 | -8.1 |
|  | 7-Methoxy obtusifolin | 1ad4 | -7.0 | 4urn | -7.3 | 5tcu | -7.0 | 4C13 | -7.4 | 5TW8 | -7.7 |
|  | Aurantioobtusin | 1ad4 | -6.8 | 4urn | -7.4 | 5tcu | -6.8 | 4C13 | -7.6 | 5TW8 | -7.8 |
|  | Emodin 3-O-rhamnoside | 1ad4 | -8.3 | 4urn | -8.6 | 5tcu | -8.8 | 4C13 | -10.6 | 5TW8 | -10.1 |
|  | Flavokermesic acid | 1ad4 | -7.1 | 4urn | -7.9 | 5tcu | -7.3 | 4C13 | -10.0 | 5TW8 | -8.6 |
|  | 1-Desmethylaurantioobtusin 2-O-hexoside | 1ad4 | -6.8 | 4urn | -7.8 | 5tcu | -7.5 | 4C13 | -9.0 | 5TW8 | -7.7 |
|  | Sennoside C | 1ad4 | -7.7 | 4urn | -8.0 | 5tcu | -7.8 | 4C13 | -9.7 | 5TW8 | -9.5 |
|  | 8-Hydroxy 6-methoxyrubiadin | 1ad4 | -7.0 | 4urn | -8.3 | 5tcu | -7.1 | 4C13 | -10.1 | 5TW8 | -7.5 |
|  | Sennoside B | 1ad4 | -8.2 | 4urn | -8.2 | 5tcu | -8.1 | 4C13 | -9.7 | 5TW8 | -9.5 |
|  | Chrysophanol | 1ad4 | -6.8 | 4urn | -8.4 | 5tcu | -7.5 | 4C13 | -10.5 | 5TW8 | -7.9 |
|  | 1-Hydroxy 6,8-dimethoxy 2-methyl 9,10-anthracenedione 3-O-hexoside | 1ad4 | -8.6 | 4urn | -8.3 | 5tcu | -9.3 | 4C13 | -9.6 | 5TW8 | -9.6 |
|  | Xanthorin | 1ad4 | -6.8 | 4urn | -8.2 | 5tcu | -7.4 | 4C13 | -10.2 | 5TW8 | -8.1 |
|  | Emodin 1-O-(6"-rhamnosyl)-hexoside | 1ad4 | -8.1 | 4urn | -7.9 | 5tcu | -7.5 | 4C13 | -8.9 | 5TW8 | -10.0 |
|  | Physcion | 1ad4 | -7.1 | 4urn | -8.1 | 5tcu | -7.2 | 4C13 | -9.7 | 5TW8 | -7.6 |
|  | Emodin | 1ad4 | -6.8 | 4urn | -7.9 | 5tcu | -7.4 | 4C13 | -10.1 | 5TW8 | -8.0 |
|  | Physcion 8-O-(6"-hexosyl)-hexoside | 1ad4 | -7.8 | 4urn | -7.8 | 5tcu | -7.4 | 4C13 | -8.5 | 5TW8 | -9.4 |
|  | Fistulic acid | 1ad4 | -6.9 | 4urn | -8.1 | 5tcu | -7.2 | 4C13 | -8.5 | 5TW8 | -8.1 |
|  | Sennoside A | 1ad4 | -8.2 | 4urn | -8.4 | 5tcu | -8.2 | 4C13 | -10.2 | 5TW8 | -9.3 |
| ***E. coli*** | 2-Hydroxyemodin | 1e8c | -7.5 | 4v53 | -8.0 | 6ntw | -7.6 | 1kzn | -8.0 | 5v7a | -8.0 |
|  | 7-Methoxy obtusifolin | 1e8c | -7.7 | 4v53 | -7.7 | 6ntw | -7.4 | 1kzn | -8.3 | 5v7a | -8.0 |
|  | Aurantioobtusin | 1e8c | -7.9 | 4v53 | -7.5 | 6ntw | -7.3 | 1kzn | -7.5 | 5v7a | -7.5 |
|  | Emodin 3-O-rhamnoside | 1e8c | -9.9 | 4v53 | -9.6 | 6ntw | -9.2 | 1kzn | -8.7 | 5v7a | -9.1 |
|  | Flavokermesic acid | 1e8c | -8.1 | 4v53 | -7.9 | 6ntw | -7.5 | 1kzn | -7.9 | 5v7a | -8.5 |
|  | 1-Desmethylaurantioobtusin 2-O-hexoside | 1e8c | -7.4 | 4v53 | -7.7 | 6ntw | -7.1 | 1kzn | -7.8 | 5v7a | -7.6 |
|  | Sennoside C | 1e8c | -9.4 | 4v53 | -8.7 | 6ntw | -9.3 | 1kzn | -8.3 | 5v7a | -8.4 |
|  | 8-Hydroxy 6-methoxyrubiadin | 1e8c | -7.9 | 4v53 | -7.2 | 6ntw | -7.0 | 1kzn | -7.8 | 5v7a | -7.4 |
|  | Sennoside B | 1e8c | -9.7 | 4v53 | -8.9 | 6ntw | -9.0 | 1kzn | -8.2 | 5v7a | -7.9 |
|  | Chrysophanol | 1e8c | -8.1 | 4v53 | -7.3 | 6ntw | -7.2 | 1kzn | -7.9 | 5v7a | -7.6 |
|  | 1-Hydroxy 6,8-dimethoxy 2-methyl 9,10-anthracenedione 3-O-hexoside | 1e8c | -9.7 | 4v53 | -9.7 | 6ntw | -9.8 | 1kzn | -8.2 | 5v7a | -9.3 |
|  | Xanthorin | 1e8c | -7.8 | 4v53 | -7.8 | 6ntw | -7.4 | 1kzn | -8.3 | 5v7a | -8.0 |
|  | Emodin 1-O-(6"-rhamnosyl)-hexoside | 1e8c | -9.2 | 4v53 | -7.9 | 6ntw | -8.2 | 1kzn | -8.0 | 5v7a | -8.6 |
|  | Physcion | 1e8c | -7.9 | 4v53 | -7.7 | 6ntw | -7.1 | 1kzn | -7.8 | 5v7a | -7.9 |
|  | Emodin | 1e8c | -7.7 | 4v53 | -7.7 | 6ntw | -7.4 | 1kzn | -8.1 | 5v7a | -7.9 |
|  | Physcion 8-O-(6"-hexosyl)-hexoside | 1e8c | -8.8 | 4v53 | -8.4 | 6ntw | -8.2 | 1kzn | -8.1 | 5v7a | -8.7 |
|  | Fistulic acid | 1e8c | -7.9 | 4v53 | -8.2 | 6ntw | -7.4 | 1kzn | -7.6 | 5v7a | -7.8 |
|  | Sennoside A | 1e8c | -9.2 | 4v53 | -9.0 | 6ntw | -9.6 | 1kzn | -8.3 | 5v7a | -8.0 |

**Table S3.** Selected protein-ligand complexes for MM/PBSA binding free energy analysis based on molecular dynamics simulations.

| **Complex** | **Frames** | **VDWAALS** | **EEL** | **EGB** | **ESURF** | **GGAS** | **GSOLV** | **TOTAL** |
| --- | --- | --- | --- | --- | --- | --- | --- | --- |
| *E. coli 30S ribosome S3_sennoside A* | Average | -25.69 | -572.36 | 577.86 | -4.74 | -598.1 | 573.12 | -24.93 |
|  | SD | 1.49 | 11.25 | 7.24 | 0.2 | 9.76 | 7.04 | 2.72 |
|  | SEM | 1.06 | 7.96 | 5.12 | 0.14 | 6.9 | 4.97 | 1.93 |
| *S. aureus 30S ribosome S3_1-Hydroxy 6,8-dimethoxy 2-methyl 9,10-anthracenedione 3-O-hexoside* | Average | -38.41 | -27.55 | 46.54 | -5.06 | -65.97 | 41.47 | -24.49 |
|  | SD | 0 | 0 | 0 | 0 | 0 | 0 | 0 |
|  | SEM | 0 | 0 | 0 | 0 | 0 | 0 | 0 |
| *E coli dihydropteroate synthase_sennoside C* | Average | -37.94 | -64.98 | 92.34 | -5.61 | -102.9 | 86.72 | -16.2 |
|  | SD | 3.8 | 16.58 | 13.72 | 0.1 | 12.78 | 13.82 | 1.04 |
|  | SEM | 2.69 | 11.73 | 9.7 | 0.07 | 9.04 | 9.77 | 0.74 |
| *E. coli Gyrase B_Senoside_A* | Average | -38.29 | 201.64 | -170.01 | -7.06 | 163.35 | -177.08 | -13.72 |
|  | SD | 3.15 | 5.47 | 6.91 | 0.25 | 2.31 | 7.16 | 4.85 |
|  | SEM | 2.23 | 3.87 | 4.88 | 0.18 | 1.64 | 5.06 | 3.43 |
| *S. aureus Gyrase B_Chrysophanol* | Average | -26.15 | -38.16 | 41.98 | -3.97 | -64.31 | 38.01 | -26.3 |
|  | SD | 0.96 | 0.26 | 1.2 | 0.09 | 0.7 | 1.1 | 0.4 |
|  | SEM | 0.68 | 0.18 | 0.85 | 0.07 | 0.5 | 0.78 | 0.28 |
| *S. aureus MurE_Chrysophanol* | Average | -30.33 | -21.96 | 32.94 | -4 | -52.29 | 28.95 | -23.34 |
|  | SD | 0.38 | 2.61 | 1.35 | 0 | 2.23 | 1.34 | 0.89 |
|  | SEM | 0.27 | 1.85 | 0.95 | 0 | 1.58 | 0.95 | 0.63 |
| *E. coli MurE_sennoside B* | Average | -39.65 | 183.67 | -173.74 | -6.91 | 144.02 | -180.65 | -36.64 |
|  | SD | 1.08 | 4.37 | 0.19 | 0.03 | 3.29 | 0.16 | 3.13 |
|  | SEM | 0.76 | 3.09 | 0.13 | 0.02 | 2.32 | 0.11 | 2.21 |
| *S. aureus Transpeptidase_Emodin 1-O-hexoside* | Average | -28.14 | -36.86 | 55.23 | -4.51 | -65 | 50.72 | -14.28 |
|  | SD | 0.53 | 3.56 | 0.05 | 0.13 | 3.03 | 0.18 | 2.85 |
|  | SEM | 0.37 | 2.51 | 0.03 | 0.09 | 2.14 | 0.13 | 2.01 |
| S. aureus Dihydropteroate synthase_Sennoside_B | Average | -21.05 | 21.86 | -7.59 | -3.1 | 0.81 | -10.7 | -9.88 |
|  | SD | 0 | 0 | 0 | 0 | 0 | 0 | 0 |
|  | SEM | 0 | 0 | 0 | 0 | 0 | 0 | 0 |
